# Microbiota Survey of Sliced Cooked Ham During the Secondary Shelf Life

**DOI:** 10.3389/fmicb.2022.842390

**Published:** 2022-03-08

**Authors:** Gloria Spampinato, Francesco Candeliere, Alberto Amaretti, Fabio Licciardello, Maddalena Rossi, Stefano Raimondi

**Affiliations:** ^1^Department of Life Sciences, University of Modena and Reggio Emilia, Modena, Italy; ^2^BIOGEST-SITEIA, University of Modena and Reggio Emilia, Modena, Italy

**Keywords:** cooked ham, secondary shelf life, food waste, *Leuconostoc carnosum*, *Leuconostoc mesenteroides*, *Carnobacterium divergens*

## Abstract

Sliced cooked ham packaged in a modified atmosphere is a popular ready-to-eat product, subjected to abundant microbial contamination throughout its shelf life that can lead to deterioration of both sensorial properties and safety. In this study, the microbial load and the chemical–physical features of cooked ham of five producers were monitored for a period of 12 days after the opening of the packages (i.e., the secondary shelf life), during which the products were stored in a domestic refrigerator at 5.2 ± 0.6°C. The sensorial properties presented a perceivable decay after 8 days and became unacceptable after 12 days. High-performance liquid chromatography analysis and solid-phase microextraction coupled with gas chromatography profiling of volatile metabolites indicated that lactic acid, ethanol, acetic acid, acetoin, 3-methyl-1-butanol, and 2-3 butanediol were the main metabolites that characterized the evolution of the analyzed cooked ham. The microbiota was monitored by 16S ribosomal RNA gene profiling and culture-dependent techniques. Already at the opening of packages, all the products presented high microbial load, generally dominated by lactic acid bacteria, with evident differences among the products. The increase of lactic acid bacteria somehow protected samples from abundant contamination by other bacteria, concurring with the evolution of more safe products. This role was exerted by numerous *Latilactobacillus*, *Leuconostoc*, and *Carnobacterium* species, among which the most frequently detected were *Latilactobacillus sakei*, *Latilactobacillus sakei carnosum*, *Leuconostoc mesenteroides*, and *Carnobacterium divergens.* Some products presented more complex communities that encompassed Proteobacteria such as *Moellerella wisconsensis*, *Proteus hauseri*, *Brochothrix thermosphacta*, and less frequently *Pseudomonas*, *Erwinia*, and *Massilia*. Opportunistic pathogenic bacteria such as *Escherichia coli* and V*ibrio* sp. were found in small quantities. The yeasts *Kazachstania servazzii* and *Debaryomyces hansenii* occurred already at 0 days, whereas various species of *Candida* (*Candida zeylanoides*, *Candida sake*, *Candida norvegica*, and *Candida glaebosa*) were abundant only after 12 days. These results indicated that the microbiological contaminants overgrowing during the secondary shelf life did not derive from environmental cross-contamination at the opening of the tray but were already present when the packages were opened, highlighting the phases of production up to the packaging as those crucial in managing the safety risk associated to this product.

## Introduction

According to the Food and Agriculture Organization, food production in the world is higher than the demand and ensues a huge amount of wasted food per year, aggravating CO_2_ emissions, land utilization, and blue water consumption (FAO, 2013; Mekonnen and Gerbens-Leenes, 2020). This phenomenon is also reflected in a series of economic, ethical, and social repercussions, considering that 11% of the world’s population has no access to food resources (Wohner et al., 2019). Unlike other food losses that may occur along the food production and supply chain, food wastes are recognized to take place in the last phases of the chain, i.e., the distribution, sale, and consumption, especially at the household level (FAO, 2013; Abdelradi, 2018; Salihoglu et al., 2018; UNEP, 2021). The assessment of appropriate secondary shelf life, defined as the time after package opening during which the food product retains a required level of quality, is pivotal for reducing domestic food waste (Nicosia et al., 2021). Indeed, excessively short secondary shelf life suggested by producers, if not relying on scientific data, may mislead consumers to discard foods still suitable for consumption.

The highest cost in wasted resources is associated with animal-based foods, which have the highest value of footprint (FAO, 2013; Mekonnen and Gerbens-Leenes, 2020). Meat waste represents a major contribution to food losses in developed countries (FAO, 2011). The large availability of food, together with the greater awareness of health and hygiene risks associated with the consumption of altered meat, means that meat products are not generally consumed beyond the expiration date or are rejected due to spoilage or minimal undesirable features perceived as defects (Iulietto et al., 2015; Ojha et al., 2020). The changes in consumers’ lifestyles have also led to the increasing demand for ready-to-eat products (Bonifacie et al., 2021). Among them, sliced cooked ham in modified atmosphere packaging (MAP) is a main product of the meat processing industry. Although cooked ham undergoes cooking at T ≥ 70°C, bacterial proliferation occurs during the whole shelf life even after maintaining hygienic precautions and preservative procedures (Raimondi et al., 2019), and a throughout survey on the sources of contamination after the cooking is still lacking.

Sliced cooked ham in MAP is subjected to abundant microbial contamination throughout production and shelf life, which leads to deterioration of both sensorial properties and wholesomeness. The microbiota of this product has been the subject of several studies, consistently indicating lactic acid bacteria (LAB) as the main contaminants that proliferate throughout the shelf life and reach remarkably high counts of 10^7^–10^9^ cfu/g (Menezes et al., 2018; Raimondi et al., 2019). Temperature, pH, water activity, nutrient availability, redox potential, and gas composition trigger microbial growth (Iulietto et al., 2015), affecting the preservation of food along the cold chain and consequently influencing the wastage (Vasilopoulos et al., 2008). LAB may concur with meat preservation due to the production of organic acids and hydrogen peroxide, which exert an intrinsic antimicrobial effect. Furthermore, selected strains produce bacteriocins specifically inhibiting certain contaminating pathogen bacteria, such as *Listeria* (Raimondi et al., 2021). On the other hand, LAB may contribute to spoilage of MAP cooked ham, inducing discoloration, changes in odor, flavor, and texture, and slime formation, negatively affecting the shelf life (Samelis et al., 2000; Kreyenschmidt et al., 2010; Raimondi et al., 2019). The spoilage of LAB is mainly caused by homofermentative bacteria belonging to the species *Latilactobacillus curvatus* and *Latilactobacillus sakei* or heterofermentative such as *Leuconostoc carnosum*, *Leuconostoc gelidum, Carnobacterium divergens*, and *Carnobacterium maltaromaticum*, whereas the Listeriaceae *Brochothrix thermosphacta* occurs when oxygen is present inside the packages (Vasilopoulos et al., 2008; Hu et al., 2009; Raimondi et al., 2019).

Some information is available on the evolution of microbial and sensorial properties of MAP cooked ham throughout the shelf life, but the evolution of the product after the opening of the package has not been investigated so far (Vasilopoulos et al., 2008; Kreyenschmidt et al., 2010; O’Neill et al., 2018; Raimondi et al., 2019). In this work, we studied the secondary shelf life of MAP cooked hams of five Italian industries, simulating the storage in a domestic fridge. Detailed information on the evolution of the products, including microbiological, sensorial, and chemical features, was collected for a period of 12 days after the opening of packages to provide the scientific basis for a more accurate recommendation of the secondary shelf life of MAP cooked ham.

## Materials and Methods

### Sample Collection and Experimental Design

Samples of MAP sliced cooked ham produced by five selected industries were purchased in local markets (Modena, Italy). For each of the five products, three packages with the same expiration date (±3 days) were purchased. This kind of product available in the Italian market has a shelf life of typically 4 weeks; all the selected packages had at least 2 weeks of remaining shelf life at the purchase time. Where reported, the producers suggest food consumption within 1–3 days after package opening. Ingredients and nutritional composition are reported in Supplementary Table 1. Microbiological, chemical, and sensorial analyses were carried out at the opening of packages (0 days), then every 4 days (4, 8, and 12 days), during which the samples were maintained in plastic bags within the same domestic refrigerator set to 4°C. The actual temperature of the domestic refrigerator was monitored and recorded using a probe and data logger (mini TH, XS Instruments, Carpi, Italy).

All chemicals were purchased from Sigma-Aldrich (St. Louis, MO, United States) unless otherwise stated.

### Sensorial Evaluation and Spoilage Assessment

At each time point, a slice of cooked ham was allowed to reach room temperature for 15 min; then, sensory analyses were carried out by a trained sensory panel. Occurring defects were evaluated through odor, texture, and color descriptors, which were given a score in the range between 0 (no defects, highest sensory quality) and 5 (highest level of defects, lowest sensory quality).

### Chemical Analyses

The pH was measured in five distinct points per sample using a puncture electrode (Sension + electrode 5,233, HACH, Switzerland). Organic acids, glucose, and ethanol were determined by high-performance liquid chromatography, according to Vasilopoulos et al. (2008). Volatile organic compounds (VOCs) were tracked by headspace solid-phase microextraction coupled with gas chromatography–mass spectrometry analysis, following the protocol described by Raimondi et al. (2018).

### Microbiological Analyses

Ten grams of cooked ham was suspended and homogenized in saline peptone water (8.5 g/L NaCl and 1 g/L peptone) at the concentration of 10% (w/v) utilizing a Lab Blender Stomacher (Seward Medical, London, United Kingdom) for 4 min. Appropriate decimal dilutions in saline peptone water were spread on agar plates for the enumeration and isolation of bacteria and fungi: plate count agar (PCA) for total aerobic bacteria, Lactobacilli MRS Agar for LAB, violet red bile glucose agar (VRBGA) for enterobacteria, and rose bengal chloramphenicol agar (RBCA) for fungi, and Baird–Parker agar (BPA) for putative staphylococci. The plates were incubated in aerobiosis at 30°C for 48 h, except for RBC plates incubated at 25°C for 5–7 days and MRS plates incubated in an anaerobic jar at 30°C for 72 h, with anaerobiosis generated by GasPaK (BD Difco). To detect *Salmonella* sp., the initial suspension was pre-enriched for 24 h at 30°C, and 100 μl was seeded in 10 ml of Rappaport–Vassiliadis soy peptone broth and incubated for 24 h at 41°C. The enrichment culture was then seeded on xylose lysine deoxycholate plates and incubated for 24 h at 37°C. All the media were purchased from BD Difco (Franklin Lake, NJ, United States).

After enumeration, a representative number of colonies (up to 48 colonies per sample per medium) were randomly picked up from PCA, MRSA, RBCA, VRBGA, and BPA and streaked in the same medium. Instagene Matrix (Bio-Rad Laboratories, Redmond, WA, United States) was used for the extraction of polymerase chain reaction (PCR) amplifiable DNA. The clones were amplified with M13-random amplified polymorphic DNA (RAPD) primer (5′-GAGGGTGGCGG TTCT-3′), and the ensuing RAPD-PCR profiles were clustered in biotypes with > 75% similarity by Pearson correlation coefficient (Quartieri et al., 2016). A single strain for each biotype was taxonomically characterized by sequencing the V1–V3 portion of the 16S ribosomal RNA (rRNA) gene for bacteria and the ITS1 for fungi. The couples of primers 16S-500f (5′-TGG AGA GTT TGA TCC TGG CTC AG-3′)/16S-500r (5′-TAC CGC GGC TGC TGG CAC-3′) and ITS1(5′-TCC GTA GGT GAA CCT TGC GG-3′)/ITS4 (5′-TCC TCC GCT TAT TGA TAT GC-3′) were utilized to amplify the two target regions, respectively (White et al., 1990; Kolbert et al., 2004). Comparisons with the reference sequences available in the GenBank database were obtained by the National Center for Biotechnology Information BLAST software^1^.

### 16S Ribosomal RNA Gene Profiling

Metataxonomic analysis was performed when packages were opened (0 days) and after 8 days, when sensorial properties were still acceptable. Two grams of cooked ham was collected and homogenized in 10 ml of food lysis buffer (DNeasy Mericon Food Kit; Qiagen, Hilden, Germany), and total DNA was extracted following the manufacturer’s standard protocol. DNA concentration was adjusted to 5 ng/μl after quantification with a Qubit 3.0 fluorimeter (Thermo Fisher Scientific, Waltham, MA, United States). Partial 16S rRNA gene sequences were performed by a DNA sequencing service provider (Eurofins Genomics, Ebersberg, Germany), which amplified and sequenced the V3–V4 region of the 16S rRNA gene using an Illumina MiSeq instrument (Illumina, San Diego, CA, United States). The sequences are available in the National Center for Biotechnology Information repository with the BioProject ID.

Raw sequences were analyzed with the QIIME 2.0 pipeline, version 2021.4 (Bolyen et al., 2019), with appropriate plugins for trimming (CUTADAPT) and denoising (DADA2) into amplicon sequence variants (Martin, 2011; Callahan, 2016). Taxonomy assignment was carried out with the feature classifier VSEARCH (Rognes et al., 2016), with SILVA SSU database release 138.1 as a reference (^2^ note that the database, despite being the latest, is not up to date with the most recent nomenclature changes in the Lactobacillaceae and Leuconostocaceae families), and similarity threshold set at 0.97. The appropriate QIIME2 plugins were utilized to compute the alpha diversity (Chao1 and Pielou’s evenness) and beta diversity (Bray–Curtis dissimilarity) and to compare them within and between 0- and 8-day samples (i.e., the Kruskal–Wallis test for alpha diversity; permutational multivariate analysis of variance for beta diversity). Differences were considered significant for *P* < 0.05. Principal coordinate analysis was computed with QIIME2, based on the beta-diversity distance matrix.

### Statistical Analysis

Unless otherwise specified, values were reported as means ± SD of triplicate samples. The comparison was performed according to Student’s *t*-test or one-way analysis of variance followed by Tukey *post hoc* comparisons, using the MaxStat software (MaxStat Pro, version 3.6).

Kruskal–Wallis test followed by Dunn’s non-parametric post-comparison test was performed on sensorial properties. Differences were considered statistically significant for *P* < 0.05.

Principal component analysis (PCA) was used to explore the data matrix of VOCs with size {60, 157}, including the 157 relative areas of VOCs, determined for the three replicates of each product at the four time points. The number of significant principal components (PCs) was defined using the screen plot, which reports the percentage of variance explained by each PC vs. the PC number. The PCA model was calculated using Past version 3.14 (Hammer et al., 2001).

Linear discriminant analysis effect size^3^ was utilized to discover distinctive taxonomic features characterizing samples at 0 and 8 days (Segata et al., 2011). Taxa presenting a significant differential abundance (*P* < 0.05) and logarithmic, linear discriminant analysis score > 2 were considered as microbial biomarkers of the corresponding timepoint.

## Results

Sliced cooked ham trays in MAP of five commercial products were stored for 12 days in a domestic refrigerator, and the temperature profile was reconstructed through a data logger. Values ranged between 4.1 and 6.5°C (Supplementary Figure 1), with a mean temperature of 5.2 ± 0.6°C (mean ± SD). The temperature fluctuated in a narrow range that was well representative of possible domestic storage without drastic temperature abuse. It should be noted that the domestic storage temperature suggested on labels is usually 0–4°C: even if the measured temperature falls out of this range, it should be considered as a realistic condition, based on previous data collected in different domestic refrigerators, which ranged from 4.6 to 10°C (Nicosia et al., 2021).

### Spoilage Assessment

**FIGURE 1:**
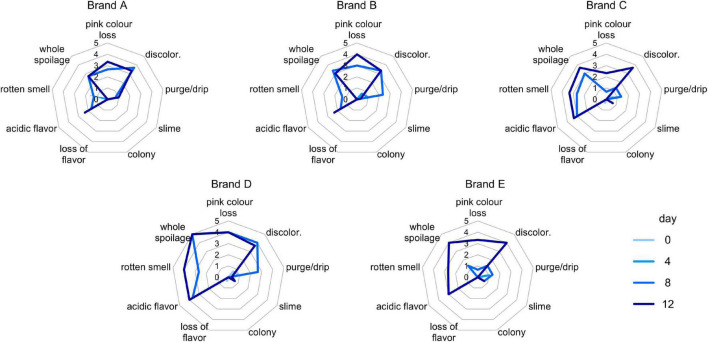
Spider charts for scores (0–5) of sensory defects detected in **(A–E)** samples, with different color lines for each timepoint.

The sensorial properties of cooked ham samples were evaluated at the time of first opening and after 4, 8, and 12 days of secondary shelf life. Generally, negative changes were recorded after 8 days, and the products became unacceptable after 12 days. The most relevant modifications were the emergence of acid flavor, rotten smell, and discoloration (Figure 1). Products behaved differently during this lapse of time. At 8 days, discoloration of D samples was significantly more pronounced than that of C and E ones (*P* < 0.05), and the overall extent of spoilage was higher in D than in other samples. At 12 days, significantly higher scores of rotten smell were registered for samples C, D, and E compared with A and B (*P* < 0.05); furthermore, the whole spoilage was higher in D than in A and B (*P* < 0.05). Samples C and D presented a reduction of drip and an increase of discoloration, rotten smell, and acidity flavor (*P* < 0.05; Supplementary Table 2).

### Culture-Dependent Enumeration

**FIGURE 2:**
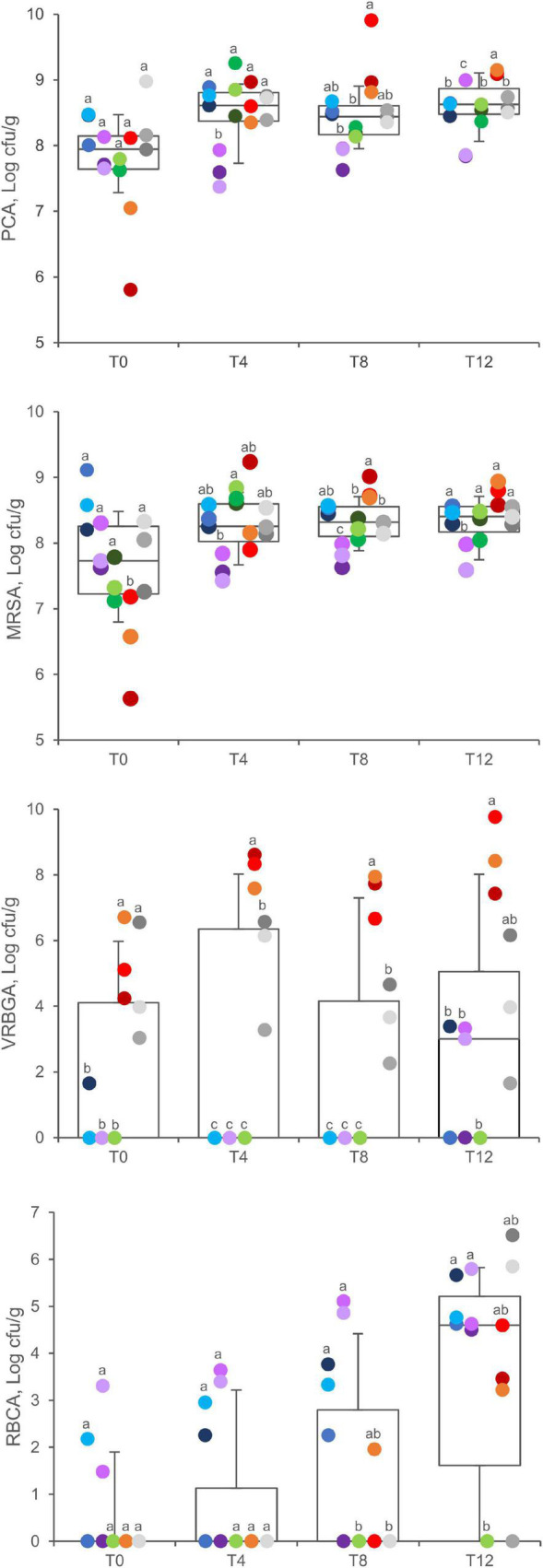
Viable counts in PCA, MRSA, VRBGA, and RBCA plates of A (blue), B (violet), C (green), D (red), and E (gray) samples. Boxes indicate 25th, 50th, and 75th percentiles; whiskers indicate 10th and 90th percentiles. Within each time point, products sharing same letter did not make a significant difference (*p* > 0.05).

Viable counts on PCA, MRSA, VRBGA, RBCA, and BPA plates were utilized to monitor total aerobic bacteria, LAB, putative enterobacteria, staphylococci, fungi, and staphylococci, respectively, throughout the secondary shelf life (Figure 2).

Total aerobic bacteria ranged between 5.8 and 9.0 Log cfu/g (7.8 ± 0.7 Log cfu/g, mean ± SD) at 0 days, without significant differences among the products (*P* > 0.05). After 4, 8, and 12 days of fridge storage, total aerobic bacteria significantly increased in packages of product D, which progressively became the most contaminated (*P* < 0.05). A significant increase was also observed for products A and C, whereas products B and E did not exhibit any significant change.

The initial charge of LAB widely differed among samples being comprised from 5.7 to 9.1 Log cfu/g (7.7 ± 0.9 Log cfu/g, mean ± SD), with product D samples presenting the lowest values (*P* < 0.01). After 4, 8, and 12 days, the spread became narrower, with LAB charges lying in the range of 7–9 Log cfu/g (mean = 8.3 Log cfu/g). Throughout the secondary shelf life, LAB significantly increased in samples of products C and D, with the latter progressively becoming the richest (*P* < 0.05). In the other products, LAB remained unchanged.

Initially, putative enterobacteria lay below the limit of detection in most of the samples of products A, B, and C, whereas all the samples of products D and E presented contamination in the range of 3.0 and 6.7 Log cfu/g, without significant differences between them (*P* > 0.05). Throughout the secondary shelf life, putative enterobacteria increased up to 9.7 Log cfu/g in D samples (*P* < 0.05), whereas they remained unchanged in E samples (*P* > 0.05). After 12 days, Enterobacteriaceae also appeared in a few samples of products A and B, which presented a load up to 3.3 Log cfu/g, whereas they were never found in product C.

Fungi were initially found only in three samples (A3, B2, and B3). In A samples, the load of fungi increased over time, reaching 5.0 ± 0.6 at T12. After 12 days, fungi were isolated from most of the samples (11 of 15), with a mean charge of 4.9 ± 1.0 Log cfu/g.

Putative staphylococci and *Salmonella* sp. were analyzed after 12 days of secondary shelf life. Putative staphylococci were found in A, D, and E samples, with counts ranging between 2.5 and 6.9 Log cfu/g (Supplementary Table 3). *Salmonella* sp. was absent in all the samples.

### Identification of the Isolates

Five hundred sixty isolates were selected from plates at 0 and 12 days and were clustered into 68 dominant biotypes, according to RAPD-PCR fingerprinting (Supplementary Table 4). One representative of each biotype was subjected to the sequencing of the 16S rRNA gene or ITS to obtain species designation.

The clones isolated in PCA belonged to 25 biotypes belonging to 11 species: *C. divergens* (5), *L. sakei* (5), *Leuconostoc mesenteroides* (5), *L. carnosum* (2), *Bacillus nealsonii* (1), *Candida zeylanoides* (1), *Carnobacterium gallinarum* (1), *C. maltaromaticum* (1), *Carnobacterium* sp. (1), *Massilia aurea* (1), *Moellerella wisconsensis* (1), and *Proteus hauseri* (1). The clones isolated in MRSA belonged to 15 biotypes, attributed to the species *L. sakei* (7), *L. mesenteroides* (3), *L. carnosum* (2), *C. divergens* (1), *L. curvatus* (1), and *Weissella viridescens* (1). Six strains were isolated from VRBGA plates, belonging to the species *Erwinia billingiae* (2), *Massilia arvi* (1), *M. wisconsensis* (1), *P. hauseri* (1), and *Pseudomonas* sp. (1). Thirteen different biotypes were found on RBCA plates, belonging to the species *C. zeylanoides* (5), *Kazachstania servazzii* (3), *Candida glaebosa* (1), *Candida novergica* (1), *Candida sake* (1), *Debaryomyces hansenii* (1), and *Debaryomyces* spp. (1). The nine biotypes were isolated on BPA plates at T12 that belonged to the species *P. hauseri* (5), *Staphylococcus xylosus* (2), *Corynebacterium stationis* (1), and *Kocuria* spp. (1).

*Leuconostoc mesenteroides* and/or *L. carnosum* abundantly contaminated the samples belonging to products A, B, C, and E, occurring already at 0 days and after 12 days in the magnitude of 7–8 Log cfu/g in PCA or MRSA plates (Table 1). In product D, a high load of *L. carnosum* (up to 8.9 Log cfu/g) appeared only after 12 days. *L. sakei* was found only in samples of products A, B, and E, being generally found in the magnitude of 6–8 Log cfu/g MRSA in plates both at 0 and 12 days. In product C, *L. sakei* occurred only at 0 days. *C. divergens* characterized only samples of products D and E, being present already at 0 days, generally in the magnitude of 7 Log cfu/g, and reaching up 9 Log cfu/g after 12 days. *C. gallinarum* and other *Carnobacterium* sp. occurred only in samples of product C, at 0 and 12 days, respectively. Interestingly, only for C samples, all the biotypes of the isolates at both 0 and 12 days were LAB. *W. viridescens* occurred only after 12 days in two samples of product D, where it reached the remarkable concentration of 9.1 Log cfu/g.

**TABLE 1:** Dominant microbial contaminants isolated from PCA (P), MRSA (M), VRBGA (V), RBCA (R), and BPA (B) plates at 0 and 12 days of secondary shelf life.

			LAB									Proteobacteria						Other bacteria				Fungi						
						
Product	Package	Sampling time (d)	*Carnobacterium divergens* (P, M)	*Carnobacterium gallinarum* (P)	*Carnobacterium maltaromaticum* (P)	*Carnobacterium* sp. (P)	*Latilactobacillus sakei* (M, P)	*Latilactobacillus curvatus* (M)	*Leuconostoc carnosum* (M, P)	*Leuconostoc mesenteroides* (M, P)	*Weissella viridescens* (M)	*Erwinia billingiae* (V)	*Massilia arvi* (V)	*Massilia aurea* (P)	*Moellerella wisconsensis* (V, P)	*Proteus hauseri* (V, B, P)	*Pseudomonas* sp. (V)	*Bacillus nealsonii* (P)	*Corynebacterium stationis* (B)	*Kocuria* sp. (B)	*Staphylococcus xylosus* (B)	*Candida glaebosa* (R)	*Candida norvegica* (R)	*Candida sake* (R)	*Candida zeylanoides* (R, P)	*Debaryomyces hansenii* (R)	*Debaryomyces* sp. (R)	*Kazachstania servazzii* (R)
A	1	0					8.2		7.2	8.4																		
		12							8.1	8.5		2.5	2.5			2.5	3.1			3.1	3.3				5.7			
	2	0					7.0		7.8	8.0																		
		12							8.4	8.6											3.0				4.6			
	3	0					8.0			8.5																2.2		
		12								8.6						2.2					2.6				4.8			
B	1	0					7.5		6.9	7.5				7.2														
		12					6.8		7.4	7.7				7.2											3.9			4.4
	2	0					8.3			8.1																		1.5
		12					7.2			8.0		3.3																4.6
	3	0					7.4		7.2	7.7																		3.3
		12					7.2			7.8		3.0																5.8
C	1	0		6.9			7.5		7.8																			
		12				8.2			8.4																			
	2	0		7.3					7.1																			
		12				8.4			8.0																			
	3	0					7.3		7.3																			
		12				8.5			8.5										2.0									
D	1	0	5.5												5.8	5.5												
		12	8.9						7.8		9.5				7.4	6.9						3.5						
	2	0	7.8		7.8											5.1												
		12	9.1						8.7		8.2				9.7	6.1						4.1	4.4					
	3	0								6.6																		
		12	8.9						8.9						8.4	6.4						2.6	3.1					
E	1	0	7.8				6.3		7.1	6.6					6.6													
		12	7.9				8.2		8.2	7.2					6.2	2.8								6.5				
	2	0	7.7						8.0	7.3					2.9	2.3												
		12	8.4				8.1	8.1	7.6	7.9					2.0		2.0	8.4										
	3	0	8.7				8.3		8.3						4.0													
		12					7.7	7.7	8.0	7.4					4.0									5.7			5.2	

*Isolates were genotyped by RAPD-PCR fingerprinting and subjected to 16S rRNA gene or ITS sequencing to obtain a taxonomic designation. Concentration, inferred from plate counts, is reported as Log cfu/g.*

Contamination with *M. wisconsensis* was observed only in D and E products, both at the opening of packages and after 12 days. At both the time points, the load of *M. wisconsensis* was very variable, ranging from 2.0 to 9.7 Log cfu/g, the highest value being observed in a sample of product D after 12 days. Other Gram-negative species (i.e., *E. billingiae*, *M. arvi*, *M. aurea*, *P. hauseri*, and *Pseudomonas* sp.) appeared sporadically in a single sample of different products and generally in low amounts (2–3 Log cfu/g).

Most of the yeast strains were isolated at 12 days, belonging to different species of the genus *Candida* (*C. glaebosa*, *C. norvegica*, *C. sake, and C. zeylanoides*); an exception was represented by *K. servazzii* that was detected in sample B since T0 reaching up to 5.8 Log cfu/g at the last time point.

### Analysis of Microbial Population by 16s Ribosomal RNA Gene Profiling

**FIGURE 3:**
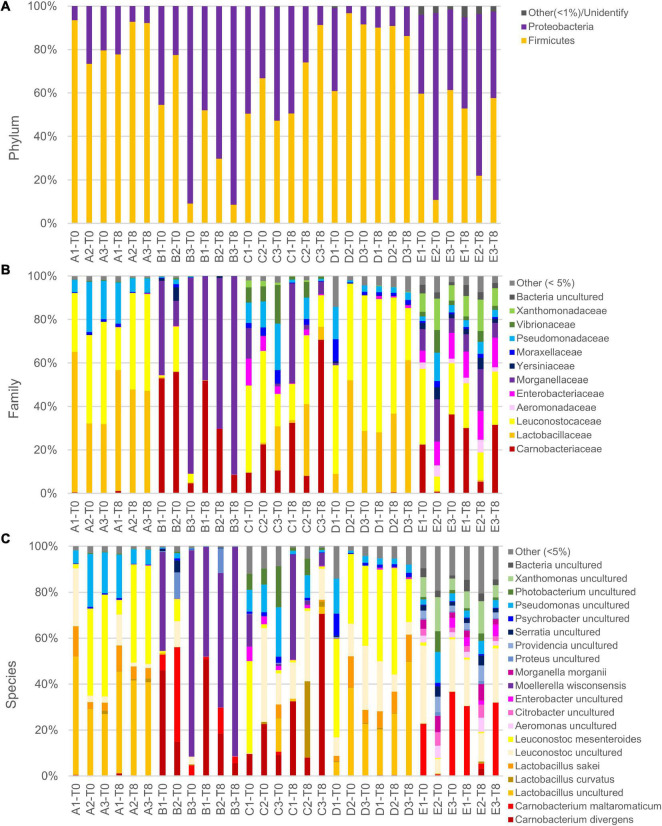
Relative abundance of main phyla **(A)**, families **(B)**, and species **(C)** in slices of cooked ham of five different products at packages opening and after 8 days. Note that in reference database, despite being latest update, Leuconostocaceae and Lactobacillaceae are reported as two separated families, but, accordingly, current nomenclature are included in sole family Lactobacillaceae. Moreover, species *Lactobacillus sakei* and *Lactobacillus curvatus* are not updated to recent name *Latilactobacillus sakei* and *Latilactobacillus curvatus.*

Metataxonomic analysis was performed at the opening and after 8 days of secondary shelf life when samples were not yet spoiled. The metagenomic survey of 16s rRNA gene profiling yielded 52,964–148,150 reads per sample, with a total of 3,442,935 in 30 samples. The reads were dereplicated into 822 amplicon sequence variants hitting a reference sequence in the Silva database and collapsed at the seventh level of taxonomic annotation into 326 operational taxonomic units. The microbiota of cooked ham was largely dominated by Proteobacteria and Firmicutes phyla (Figure 3). Chao1 richness was different among the diverse products, being highest for C samples and lowest for A and D ones (Supplementary Figure 2A). Only in D samples, richness significantly decreased after 8 days of conservation. Pielou evenness presented significant differences among the samples and showed similar levels of diversity within the same product, excluding relevant variations during the secondary shelf life (Supplementary Figure 2B). In evenness, samples of product D collected at both 0 and 8 days showed the greatest variability.

**FIGURE 4:**
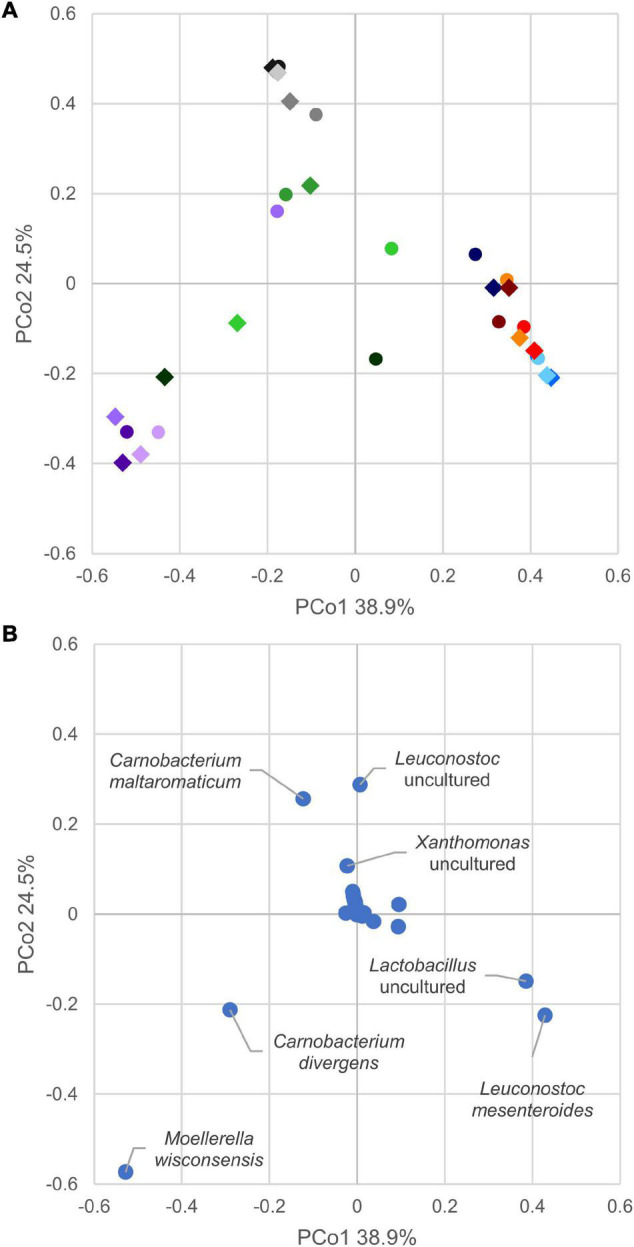
**(A)** Principal coordinate analysis plot of beta diversity based on Bray–Curtis dissimilarity index of cooked ham microbiome. Different colors indicate products, A (blue), B (violet), C (green), D (red), and E (gray). Circles mark samples at opening, diamond after 8 days. **(B)** Principal coordinate analysis plot of species contribution to sample differentiation.

In the principal coordinate analysis plot computed from the Bray–Curtis beta diversity distance matrix (Figure 4A), initial samples could not be clearly separated from those with 8 days of secondary shelf life. On the other hand, samples clustered based on the product, regardless of the timepoint. Samples of products A and D clustered together and shared some biomarkers such as *Lactobacillus* (comprising *Latilactobacillus*) and *L. mesenteroides* that mainly contributed to PCo1 (Figure 4B). Accordingly, A and D samples were dominated by Firmicutes, mainly ascribed to the families of Leuconostocaceae and Lactobacillaceae, currently reclassified into a single-family named Lactobacillaceae (Figure 3).

Generally, the evolution of the 15 samples during the first 8 days of secondary shelf life did not determine relevant changes, with a few exceptions (B2, C1, and C3). The microbiota composition of A, D, and E products was minimally perturbed over the first 8 days of secondary shelf life.

B samples lay contiguously at low PCo1 and PCo2 values, characterized by *M. wisconsensis* and *C. divergens*. Consistently, in five of six B samples, *M. wisconsensis* outnumbered the other bacteria.

E samples clustered together (high PCo1 and low PCo2 values), with *C. maltoaromaticum* and *Leuconostoc* as major biomarkers. Accordingly, the families Leuconostocaceae (currently comprised in Lactobacillaceae), Carnobacteriaceae, and Morganelaceae dominated E samples (Figure 3). Other common bacteria identified in all E samples were *Lactobacillus* (comprising *Latilactobacillus*), *Pseudomonas*, *Psychrobacter, Photobacterium*, *Xanthomonas*, *Proteus*, *Serratia*, *Providencia*, *Citrobacter*, *Aeromonas*, *Shewanella*, *Acinetobacter*, *Vibrio, Hafnia-Obesumbacterium*, *Klebsiella*, *Brochothrix*, and *Halomonas.*

Linear discriminant analysis effect size algorithm was utilized to discover distinctive taxonomic features characterizing samples at 0 or 8 days. Only Bacillaceae, *Geobacillus*, and *Parageobacillus thermoglucosidasius* exhibited significantly higher abundances in samples at 0 days (Supplementary Figure 3). The same analysis, applied to identify biomarkers specific for each product, indicated that product B was characterized by *L. mesenteroides*, product D by Enterobacterales and Morganellaceae, and product E by *Photobacterium*.

### Glucose and Organic Acids

**FIGURE 5:**
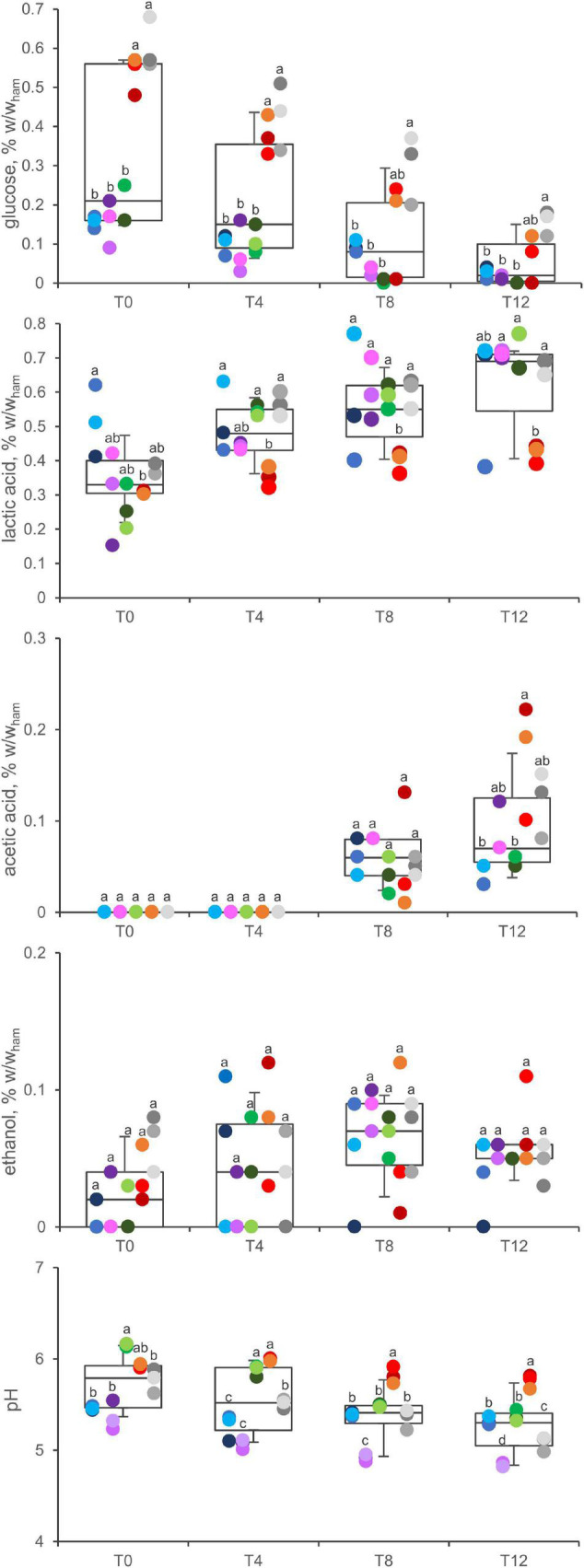
Concentration of glucose, acetic acid, lactic acid, ethanol, and pH variation in A (blue), B (violet), C (green), D (red), and E (gray) samples. Boxes indicate 25th, 50th, and 75th percentiles; whiskers indicate 10th and 90th percentiles. Within each time point, products sharing same letter did not make a significant difference (*p* > 0.05).

Glucose initially ranged from 0.09 to 0.68% w/w (g *per* 100 g of ham) (Figure 5). Its concentration was significantly higher in D and E samples than in A, B, and C ones. Overall, it significantly decreased during the secondary shelf life (*P* < 0.05). However, after 12 days of refrigeration at 5°C and relevant metabolic activity, it was still present in most samples (11 of 15) although in negligible amounts (0.01 to 0.17% w/w).

Lactic acid was the main metabolite, occurring in all the samples and at all the timepoints throughout the secondary shelf life. The initial concentration ranged from 0.15 to 0.62% w/w, with the highest levels in product A samples (*P* < 0.05). Lactic acid significantly increased during the secondary shelf life in B, C, and E samples (*P* < 0.05), where its concentration almost doubled (from 0.30 ± 0.14 to 0.71 ± 0.01; from 0.26 ± 0.07 to 0.70 ± 0.06; from 0.38 ± 0.02 to 0.68 ± 0.02 in B, C, and E, respectively), whereas it did not significantly increase in A and D samples.

Acetic acid was another major metabolite that accumulated in cooked ham over time. In all the samples, its concentration was under the limit of detection at both 0 and 4 days, whereas it accumulated toward the end of the secondary shelf life. Ethanol was detected in minor amounts, without significant differences among the products, generally tending to accumulate over time.

The pH of cooked ham decreased progressively during the secondary shelf life (5.7 ± 0.3 at T0; 5.5 ± 0.4 at T4; 5.4 ± 0.3 at T8; 5.3 ± 0.3 at T12; overall means ± SD) (*P* < 0.01), with different kinetics depending on the product.

### Volatile Organic Compounds

In the headspace of cooked ham samples, solid-phase microextraction coupled with gas chromatography analysis revealed a total of 157 VOCs, including organic acids, ethanol, and other alcohols, ketones, fatty aldehydes, esters, phenols, and a variety of aromatic compounds, furans, linear and branched aliphatic hydrocarbons, terpenoids, and sulfides (Supplementary Figure 4).

**FIGURE 6:**
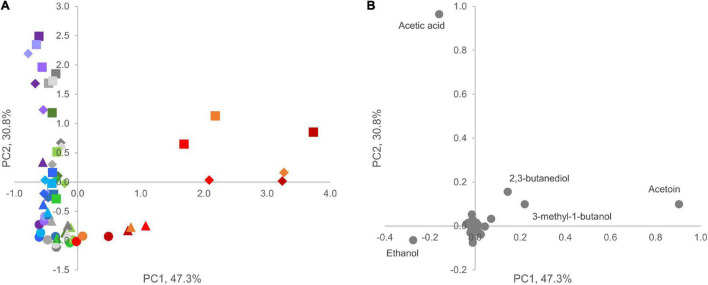
Principal component analysis plot (*PC1* vs. PC2) of VOC profiles in A (blue), B (violet), C (green), D (red), and E (gray) samples. **(A)** Score plot of cooked ham samples; circles, triangles, diamonds, and squares indicate different sampling times (0, 4, 8, and 12 days, respectively). **(B)** Loading plot of volatile molecules. Compounds with highest loading value in PC1 vs. PC2 are labeled.

In the PCA model of VOC profiles, the two most informative dimensions, PC1 and PC2, accounted for 47 and 31% of data variability, respectively (Figure 6A). Ethanol, acetic acid, and acetoin presented a major loading in determining differences among samples throughout the secondary shelf life. Acetoin, 3-methyl-1-butanol, and 2-3 butanediol presented a positive contribution to PC1, whereas ethanol and acetic acid presented a negative one (Figure 6B). Interestingly, only product D was characterized by higher values of PC1 compared with other products, but differences became evident only during the secondary shelf life, with increasing PC1 values over time. Acetic acid was the main positive contributor to PC2. All samples at 0 days were grouped at low values of PC1 and PC2, regardless of the product. A common trend of all products was the accumulation of acetic acid: the distribution of samples along the PC2 clearly showed a correlation between increasing time from the opening of the trays and increasing values of PC2.

## Discussion

Modified atmosphere packaging sliced cooked ham is a popular ready-to-eat product that has been the subject of several studies aiming to define its microbiota and the deriving impact on safety, spoilage, and preservation (Vasilopoulos et al., 2010; Raimondi et al., 2019; Zagdoun et al., 2020; Candeliere et al., 2021). In this study, the microbial load and the chemical–physical characteristics of cooked ham were monitored for a period of 12 days after the opening of the packages, during which the products were stored in a domestic refrigerator. All the products were purchased, opened, and analyzed in a range of 10–15 days before the expiration date. During the secondary shelf life at a mean temperature of 5.2 ± 0.6°C, the sensorial analyses of five products of MAP sliced cooked ham revealed a perceivable decay of quality after 8 days, but products became unacceptable only after 12 days, with the comparison of acid flavor, rotten smell, and discoloration. The organoleptic properties of the samples during the secondary shelf life suggest that cooked ham remains sensorially acceptable far beyond the 1–3 days suggested by the producers (Supplementary Table 1). However, the composition of the spoiling microbiota raised some concerns. The absence of viable *Salmonella* sp. in 10 g of the product after 12 days of storage is satisfying; nevertheless, the recurrent or sporadic presence of other opportunistic pathogens, such as *E. coli*, V*ibrio* sp., and *Klebsiella pneumoniae*, was identified by culture-independent methods and had relevant implications on the hygiene and safety of the products. Both metagenomic analysis and identification of the isolates revealed that, even at the opening of packages, all the products presented high microbial load and a quite rich microbiota, generally dominated by LAB. This result is in agreement with data reported in the literature indicating the genera *Leuconostoc*, *Carnobacterium*, and *Latilactobacillus* as among the main colonizers of MAP and vacuum-packed cooked ham (Vasilopoulos et al., 2010; Iskandar et al., 2017; Raimondi et al., 2019; Candeliere et al., 2021).

The 16S rRNA gene profiles were analyzed at the opening of packages and after 8 days of storing before samples became spoiled. The differences among microbiota were evident and mainly ascribed to the product, with LAB representing the prevailing group of bacteria in samples A and D, whereas more complex communities, also encompassing several Proteobacteria, were identified in the others. For instance, the Enterobacteriaceae *M. wisconsensis*, a facultative anaerobic Gram-negative rod, was the major colonizer of B samples already at 0 days. This species is probably an environmental contaminant, as literature reports a variety of isolation sources, including water, foods, and samples of human origin (Leroy et al., 2016). *M. wisconsensis* can be involved in the dissemination of antibiotic resistance, and its pathogenic role is debated (Stock et al., 2003; Leroy et al., 2016; Ahmad et al., 2020).

Culture-based identification revealed the presence of another contaminant, Enterobacteriaceae, *P. hauseri*. It is a Gram-negative motile bacterium, related to the human pathogens *Proteus vulgaris* and *Proteus mirabilis*, which was recurrent within the packages of products A and D, where it increased to remarkably high levels of contamination at 12 days. Other Proteobacteria, belonging to the genera *Pseudomonas*, *Erwinia*, and *Massilia*, occurred less frequently and abundantly. Facultative anaerobic Gram-negatives evidently contaminated the product at some level during the processing and before the packaging and, like LAB, grew in the ham unhindered by the modified atmosphere (Stock et al., 2003; Kreyenschmidt et al., 2010; Drzewiecka, 2016). *S. xylosus* occurred only in product A. It is used as a biocontrol agent to preserve meat products from mycotoxin produced by fungi, can convert nitrates to nitrites, and is responsible for aroma components (Stahnke, 1994; Cebrián et al., 2020).

The metagenomic analysis pointed out the occurrence of other bacterial contaminants that were not retrieved with the culture-dependent method and that could represent a potential concern for sensorial properties and/or food safety if they overgrew. *B. thermosphacta*, which is reported to give cooked ham sour off-flavors, discoloration, and slime (Borch et al., 1996; Vasilopoulos et al., 2015; Dušková et al., 2016; Raimondi et al., 2019), was found in almost all the samples already at the beginning of the secondary shelf life, although in low amounts (up to 1.1%). Opportunistic pathogenic bacteria were also identified, even if in small quantities. *E. coli* and V*ibrio* sp. were other frequent contaminants, being found in most of the samples, reaching up to 3.0 and 1.8% of the microbiota, respectively (means 0.5 and 0.2%, respectively), whereas *Salmonella enterica* and *K. pneumoniae* occurred sporadically in a sole or few samples.

The presence of *K. servazzii* and *D. hansenii* already at 0 days confirms these yeast species as occasional contaminants of cooked meat products (Hernández et al., 2018), which colonized the products before the packaging and thrived during the secondary shelf life; indeed, once the packages have been opened, the protective effect of the modified atmosphere is lost, with only a slight residual inhibitory activity of the CO_2_ solubilized in the matrix. Yeast strains belonging to the genus *Candida* (*C. zeylanoides*, *C. sake*, *C. norvegica*, and *C. glaebosa*) lay below the limit of detection at the opening of packages but were abundantly found after 12 days. Although it could not be excluded that *Candida* spp. contaminated the cooked ham during the secondary shelf life, the appearance of *C. sake*, *C. glaebosa*, and *C. zeylanoides* seemed associated with specific products and could originate from the manufacturing. Frequent sources of yeast contamination are the surfaces of machinery entering in contact with meat during the processing (Hernández et al., 2018). The growth of yeasts is undesirable in meat products, as it causes off-flavors, discoloration, and the formation of slime (Nielsen et al., 2008).

Both culture-dependent and independent characterization of the spoiling microbiota suggested that the high load of bacteria already present at the opening of cooked ham packages prevented the bloom of other species and determined the outbreak of dominant bacteria. For example, a further increase of LAB was observed in the samples where they were already abundant at the opening of packages, leading *Leuconostoc*, *Carnobacterium*, *Latilactobacillus*, and, to a lower extent, *Weissella* to dominate these microbial communities. On the other hand, the growth of Gram-negatives and other bacteria occurred in samples initially characterized by a complex community, where LAB was not dominant. The increase of LAB somehow protected the samples from abundant contamination by other bacteria, concurring to the evolution of more safe products. In these samples, a similar role was exerted by several *Latilactobacillus*, *Leuconostoc*, and *Carnobacterium* species, among which the most frequently detected were *L. sakei*, *L. carnosum*, *L. mesenteroides*, and *C. divergens*. Generally, the low temperatures of storage made *L. carnosum* and *L. mesenteroides* the species best adapted to cooked ham (Vasilopoulos et al., 2008; Raimondi et al., 2021). Besides their preservative effect, LAB can also be involved in spoilage (Comi et al., 2016; Raimondi et al., 2019). *L. mesenteroides*, *L. carnosum*, and *C. divergens* were claimed as responsible for greenish color around the cooked ham slices, discoloration, and off-odor, with *L. carnosum* and *L. mesenteroides* mainly in charge for sour odor (Björkroth et al., 1998; Leisner et al., 2007; Diez et al., 2008; Schirmer et al., 2009; Comi et al., 2016).

The aroma of cooked ham is characterized by molecules obtained by thermally-induced lipid oxidation, such as straight-chain fatty acids, aldehydes, ketones, and alcohols (1-hexanol) (Leroy et al., 2009). The spices added to the product also contributed with terpenoids and sulfides such as 3-carene, gamma-terpinene, humulene, d-limonene, beta-phellandrene, and alpha-pinene (Rux et al., 2019). Moreover, during shelf life, several VOCs were produced from the catabolism of sugars, amino acids, and lipids by the growing microorganisms that dominated the microbiota of the cooked ham during storage (Estévez et al., 2003; Rivas-Cañedo et al., 2009; Holm et al., 2013; Casaburi et al., 2015; Raimondi et al., 2019). Lactic acid, ethanol, acetic acid, acetoin, 3-methyl-1-butanol, and 2-3 butanediol were the main metabolites that characterized the evolution of the analyzed cooked ham during the secondary shelf life, impacting their sensorial properties. Lactic and acetic acids, together with ethanol, were produced by LAB and other bacteria *via* saccharolytic metabolism (Casaburi et al., 2015). The increase of acetic acid as a function of sampling time was justified by the widespread presence of heterofermenting bacteria such as *L. mesenteroides* and *L. carnosum*, which dominated the spoilage microbiota of all samples. The lower amount of acetic acid compared with lactic acid was consistent with the results obtained by Vasilopoulos et al. (2008). Other compounds, such as acetoin, 3-methyl-1-butanol, 2-3 butanediol, 3-methyl-butanal, 2-butanone, 2-methyl-1-propanol, and 1-hexanol, were produced by LAB and enterobacteria. In particular, the alcohols 2-methyl-1-propanol and 3-methyl-1-butanol derive from the catabolism of amino acids of valine and leucine (Ardö, 2006). The esters were principally produced by enterobacteria and microorganisms able to form esterase involved in the formation of ethyl bonds (Argyri et al., 2015). The increased amount of acetoin in the samples was in good agreement with the complexity of isolated bacteria; in particular, the presence of *W. viridescens* and *L. carnosum* was already correlated with acetoin concentration in fermented foods (Diez et al., 2009; Candeliere et al., 2021). In stored sample D, the presence of isolated bacteria ascribable to the genus *Candida* and in particular *C. glaebosa*, *C. norvegica*, and *C. zeylanoides* was peculiar. The latter was previously described as a high-producing species of acetoin among the non-*Saccharomyces* yeast (Tofalo et al., 2014).

## Conclusion

The main causes of microbial contamination of cooked ham originate from incorrect cooking of meat, improper sanitization practices, and recontamination during slicing and packaging, ensuing the increase of contaminants during the shelf life. Consistently, the analyzed cooked ham presented a more or less rich microbiota already at the opening of packages. Modified atmosphere packaging conditions, usually consisting of 70% N_2_ and 30% CO_2_, guarantee to some extent the inhibition of chemical and microbial spoilage of the packaged product, thus ensuring a primary shelf life of at least 1 month; the package opening causes a sudden variation of the headspace gas composition and a loss of protection: this event makes so that the secondary shelf life is significantly shorter than primary shelf life. It could be speculated that the problems of microbiological contamination did not derive from conservation during the secondary shelf life but were already present when the packages were opened, 2 weeks before the expiration date of the products, indicating the phases of production up to the packaging as those crucial in managing the safety risk. A high load of LAB already present at the opening of cooked ham packages inhibited the bloom of other species and likely prevented the outbreak of pathogenic bacteria. The exploitation of specially selected strains belonging to the genera *Latilactobacillus* and *Leuconostoc* with reduced impact on the organoleptic properties of the product could be an effective strategy to control these microbial communities and extend the primary and secondary shelf life of the product, reducing domestic food losses.

## Data Availability Statement

The datasets presented in this study can be found in online repositories. The names of the repository/repositories and accession number(s) can be found below: https://www.ncbi.nlm.nih.gov/, PRJNA778960.

## Author Contributions

SR and MR conceived the study. GS and FL carried out chemical analysis. GS and SR carried out the microbiological analysis. AA and FC performed the 16S rRNA gene profiling, bioinformatics, and statistical analysis. SR wrote the manuscript with contributions from GS, FC, AA, FL, and MR. All authors contributed to the article and approved the submitted version.

## Conflict of Interest

The authors declare that the research was conducted in the absence of any commercial or financial relationships that could be construed as a potential conflict of interest.

## Publisher’s Note

All claims expressed in this article are solely those of the authors and do not necessarily represent those of their affiliated organizations, or those of the publisher, the editors and the reviewers. Any product that may be evaluated in this article, or claim that may be made by its manufacturer, is not guaranteed or endorsed by the publisher.

## Footnotes

1
https://blast.ncbi.nlm.nih.gov

2
https://www.arb-silva.de/download/arb-files/

3
http://huttenhower.sph.harvard.edu/galaxy
