# Supplementary material for: Microbiota Survey of Sliced Cooked Ham During the Secondary Shelf Life
Source: Front Microbiol. 2022 Mar 8;13:842390. doi: 10.3389/fmicb.2022.842390 (PMC8957903; doi:10.3389/fmicb.2022.842390)
Supplement: Supplementary file 1 [file Data_Sheet_1.PDF]

Microbiota Survey of Sliced Cooked Ham During the Secondary Shelf Life

Gloria Spampinato[1], Francesco Candeliere[1], Alberto Amaretti[1,2], Fabio Licciardello[1,2], Maddalena Rossi[1,2], and Stefano Raimondi[1,*]

[1] Department of Life Sciences, University of Modena and Reggio Emilia, Modena, Italy

[2] BIOGEST-SITEIA, University of Modena and Reggio Emilia, Modena, Italy
*** Correspondence:**
Stefano Raimondi
stefano.raimondi@unimore.it

Supplementary Material

Supplementary Table 1. Nutritional composition and list of the ingredients corresponding to the 5 products of sliced cooked ham in MAP retrieved from the local market.

**Product**	A	B	C	D	E
**Nutrition facts (g/100g)**					
Total Fat	6.0	16.0	6.0	2.0	2.0
of wich Saturated	2.8	6.4	1.2	0.8	0.8
Total Carbohydrate	1.1	< 0.5	0.5	1.0	1.1
of wich Sugars	0.5	< 0.6	0.5	1.0	1.1
Protein	23.0	16.0	18.0	20.0	20.0
Salt	2.3	1.8	2.0	2.0	1.9
**Ingredints**					
	pork leg	pork leg	pork meat	pork leg	pork leg
	salt	salt	salt	salt	salt
	dextrose	dextrose	dextrose	dextrose	dextrose
	sucrose	sucrose			
		spices			
	flavors	natural flavors	natural flavors	natural flavors	flavors
	Sodium ascorbate (E301)	Sodium ascorbate (E301)	Sodium ascorbate (E301)	Sodium ascorbate (E301)	Sodium ascorbate (E301)
	Sodium nitrate (E250)	Sodium nitrate (E250)	Sodium nitrate (E250)	Sodium nitrate (E250)	Sodium nitrate (E250)
**Suggested Storage Temperature (°C)**	0 - 4	1 - 4	0 - 4	0 - 4	0 - 4
**Suggested Secondary Shelf life (days)**	1	-	1	2 - 3	2 - 3

Supplementary Table 2. Sensory defects detected at the time of opening (T0) and after 4, 8 and 12 d of opening (T4, T8, T12) in the 5 tested products of cooked ham. Each value is given as mean of three replicates. At each time point, common lowercase letters in the same line indicate non significant differences between the products; common uppercase letters in the same line indicate non significant differences of a single product comparing all timepoints ($p < 0.05$). If letters are not provided, no significant difference was observed.

	T0					T4					T8					T12				
**Product**	**A**	**B**	**C**	**D**	**E**	**A**	**B**	**C**	**D**	**E**	**A**	**B**	**C**	**D**	**E**	**A**	**B**	**C**	**D**	**E**
**Pink colour loss**	0	0	0	0	0	0	0	0	0	0	2.7	3	0.7	4	0.7	3.3	4	2.3	4	3.3
**Discolor.**	0	0.7	0.3^{AB}	0.7^{A}	0	0	0.7	0^{B}	0^{A}	0	3.7^{ab}	3.3^{ab}	1.3^{aAB}	4^{bB}	1.3^{a}	3.3	3.3	3.7^{A}	3.7^{B}	4
**Purge/drip**	1	1	1^{A}	1^{A}	1	0.3	1	1^{A}	1^{A}	1	0.7	2.3	1.3^{A}	2.7^{B}	1.3	1	0.3	0^{B}	0.3^{A}	0
**Slime**	0	0	0	0	0	0	0	0	0	0	0	0	0	0	0.7	0	0	0.7	0.7	0.7
**Colony**	0	0	0	0	0	0	0	0	0	0	0	0	0	0	0	0	0	0	0	0
**Loss of flavor**	0	0	0.3	0.3	0	0	0	0	0	0	0	0	0	0	0	0	0	0	0	0
**Acidic flavor**	0	0	0^{A}	0^{A}	0	0	0	0^{A}	0.3^{A}	0	1.7	1.7	3^{B}	3.7^{B}	2	2.3	2.3	3.3^{B}	4^{B}	3
**Rotten smell**	0	0	0	0^{A}	0	1.5	0	0	0^{A}	0	1.3	1.3	2.7	2.7^{B}	0	0^{a}	0^{a}	3.3^{b}	4^{bB}	2.7^{b}
**Whole spoilage**	0	0	0	0	0	0	0	0	0	0	2.7^{a}	3.3^{a}	3^{a}	5^{b}	1.3^{a}	2.7^{a}	3^{a}	3.7^{ab}	5^{b}	4^{ab}

Supplementary Table 3. Enumeration of putative Staphylococci on BPA plate at 12 d.

Product	A	B	C	D	E
**BPA, Log cfu/g**					
Sample 1	2.5	< 2	< 2	6.9	2.8
Sample 2	3.0	< 2	< 2	6.2	< 2
Sample 3	2.8	< 2	< 2	6.4	< 2

Supplementary Table 4. List of the 69 isolates representative of the different RAPD-PCR clusters. Taxonomical characterization by partial 16S rRNA gene or ITS1 sequencing is reported.

Strain ID	Product (tray)	Sample	Medium	Sequence	bp	% identity	Query coverage	Name	Accession n°
5	A1	T0	MRSA	GCGTGCCTAATACATGCAAGTCGAACGCACTCTCGTTTAGATTGAAGGAGCTTGCTCCT<br>GATTGATAAACATTTGAGTGAGTGGCGGACGGGTGAGTAACACGTGGGTAACCTGCCC<br>TAAAGTGGGGGATAACATTTGGAAACAGATGCTAATACCGCATAAAACCTAACACCGC<br>ATGGTGTAGGGTTGAAAGATGGTTTCGGCTATCACTTTAGGATGGACCCGCGGTGCATT<br>AGTTAGTTGGTGAGGTAAAGGCTCACCAAGACCGTGATGCATAGCCGACCTGAGAGGG<br>TAATCGGCCACACTGGGACTGAGACACGGCCCAGACTCCTACGGGAGGCAGCAGTAGG<br>GAATCTTCCACAATGGACGAAAGTCTGATGGAGCAACGCCGCGTGAGTGAAGAAGGTT<br>TTCGGATCGTAAAACTCTGTTGTTGGAGAAGAATGTATCTGATAGTAACTGATCAGGTA<br>GTGACGGTATCCAACCAGAAAGCCACGGCTAACTACGTGCCAGCAGCCGCGGTAATAC<br>GTAGGTGGCAAGCGTTGTCCGGATTTATTGGGCGTAAAGCGAGCGCAGGCGGTTTCTT<br>AAGTCTGATGTGAAAGCCTTCGGCTCAACCGAAGAAGTGCATCGGAAACTGGGAAACT<br>TGAGTGCAGAAGAGGACAGTGGAACTCCATGTGTAGCGGTGAAATGCGTAGATATATG<br>GAAGAACACCAGTGGCGAAGGCGGCTGTCTGGTCTGTAACTGACGCTGAGGCTCGAAA<br>GCATGGGTAGCAAACAGGATTAGATACCCTGGTAGTCCATGCCGTAAACGATGAGTGC<br>TAGGTGTTGGAGGGTTTCCGCCCTTCAGTGCCGCAGCTAACGCATTAAGCACTCCGCCT<br>GGGGAGTACGACCGCAAGGTTGAAACTCAAAGGAATTGACGGGGGCCCGCACAAGCG<br>GTGGAGCATGTGGTTTAATTCGAAGCAACGCGAAGAACCTTACCAGGTCTTGACATCC<br>TTTGACCACTCTAGAGATAGAGCTTTCCCTTCGGGGACAAAGTGACAGGTGGTGCATG<br>GTTGTCGTCAGCTCGTGTCGTGAGATGTTGGGTTAAGTCCCGCAACGAGCGCAACCCTT<br>ATTACTAGTTGCCAGCATTTAGTTGGGCACTCTAGTGAGACTGCCGGTGACAACCGGA<br>GAAG	1168	99.8%	100%	*Latilactobacillus sakei*	NR_113821.1
8	A1	T0	MRSA	CGTGCCTAATACATGCAAGTCGAACGCACTCTCGTTTAGATTGAAGGAGCTTGCTCCTG<br>ATTGATAAACATTTGAGTGAGTGGCGGACGGGTGAGTAACACGTGGGTAACCTGCCCT<br>AAAGTGGGGGATAACATTTGGAAACAGATGCTAATACCGCATAAAACCTAACACCGCA<br>TGGTGTAGGGTTGAAAGATGGTTTCGGCTATCACTTTAGGATGGACCCGCGGTGCATTA<br>GTTAGTTGGTGAGGTAAAGGCTCACCAAGACCGTGATGCATAGCCGACCTGAGAGGGT<br>AATCGGCCACACTGGGACTGAGACACGGCCCAGACTCCTACGGGAGGCAGCAGTAGG<br>GAATCTTCCACAATGGACGAAAGTCTGATGGAGCAACGCCGCGTGAGTGAAGAAGGTT<br>TTCGGATCGTAAAACTCTGTTGTTGGAGAAGAATGTATCTGATAGTAACTGATCAGGTA<br>GTGACGGTATCCAACCAGAAAGCCACGGCTAACTACGTGCCAGCAGCCGCGGTAATAC<br>GTAGGTGGCAAGCGTTGTCCGGATTTATTGGGCGTAAAGCGAGCGCAGGCGGTTTCTT<br>AAGTCTGATGTGAAAGCCTTCGGCTCAACCGAAGAAGTGCATCGGAAACTGGGAAACT<br>TGAGTGCAGAAGAGGACAGTGGAACTCCATGTGTAGCGGTGAAATGCGTAGATATATG<br>GAAGAACACCAGTGGCGAAGGCGGCTGTCTGGTCTGTAACTGACGCTGAGGCTCGAAA<br>GCATGCGTAGCAAACAGGATTAGATACCCTGGTAGTCCATGCCGTAAACGATGAGTGC<br>TAGGTGTTGGAGGGTTTCCGCCCTTCAGTGCCGCAGCTAACGCATTAAGCACTCCGCCT<br>GGGGAGTACGACCGCAAGGTTGAAACTCAAAGGAATTGACGGGGGCCCGCACAAGCG<br>GTGGAGCATGTGGTTTAATTCGAAGCAACGCGAAGAACCTTACCAGGTCTTGACATCC<br>TTTGACACTCTAGAGATAAA	1008	99.8%	99%	*Latilactobacillus sakei*	NR_113821.1
10	A1	T0	MRSA	GTGCCTAATACATGCAAGTCGAACGCACTCTCGTTTAGATTGAAGGAGCTTGCTCCTGA<br>TTGATAAACATTTGAGTGAGTGGCGGACGGGTGAGTAACACGTGGCGTAACCTGCCCT<br>AAAGTGGGGGATAACATTTGGAAACAGATGCTAATACCGCATAGAACT	165	98.8%	99%	*Latilactobacillus sakei*	NR_113821.1
289	A1	T0	PCA	CTATACATGCAAGTCGAACGCACAGCGAAAGGTGCTTGCACCTTTCAAGTGAGTGGCG<br>AACGGGTGAGTAACACGTGGACAACCTGCCTCAAGGCTGGGGATAACATTTGGAAACA<br>GATGCTAATACCGAATAAAACTTAGTGTCGCATGACACAAAGTTAAAAGGCGCTTCGG<br>CGTCACCTAGAGATGGATCCGCGGTGCATTAGTTAGTTGGTGGGGTAAAGGCCTACCA<br>AGACAATGATGCATAGCCGAGTTGAGAGACTGATCGGCCACATTGGGACTGAGACACG<br>GCCCAAACTCCTACGGGAGGCTGCAGTAGGGAATCTTCCACAATGGGCGAAAGCCTGA<br>TGGAGCAACGCCGCGTGTGTGATGAAGGCTTTCGGGTCGTAAAGCACTGTTGTATG	404	100.0%	99%	*Leuconostoc mesenteroides*	NR_040817.1

Strain ID	Product (tray)	Sample	Medium	Sequence	bp	% identity	Query coverage	Name	Accession n°
292	A1	T0	PCA	CGTGCCTAATACATGCAAGTCGAACGCACAGCGAAAGGTGCTTGCACCTATTCAAGTG<br>AGTGGCGAACGGGTGAGTAACACGTGGACAACCTGCCTCAAGGCTGGGGATAACATTT<br>GGAAACAGATGCTAATACCGAATAAAACTTAGTGTCGCATGACACAAAGTTAAAAGGC<br>GCTTCGGCGTCACCTAGAGATGGATCCGCGGTGCATTAGTTAGTTGGTGGGGTAAAGG<br>CCTACCAAGACAATGATGCATAGCCGAGTTGAGAGACTGATCGGCCACATTGGGACTG<br>AGACACGGCCCAAACTCCTACGGGAGGCTGCAGTAGGGAATCTTCCACAATGGGCGAA<br>AGCCTGATGGAGCAACGCCGCGTGTGTGATGAAGGCTTTCGGGTCGTAAAGCACTGTT<br>GTATGGGAAGAACAGCTAGAATAGGAAATGATTTTAGTTTGACGGTACCATACCAGAA<br>AGGGACGGCTAAATACGTGCCAGCAGCCGCGGTAATACGTATGTCCCGAGCGTTATCC<br>GGATTTATTGGGCGTAAAGCGAGCGCAGACGGTTTATTAAGTCTGATGTGAAAGCCCG<br>GAGCTCAACTCCGGAATGGCATTGGAAACTGGTTAACTTGAGTGCAGTAGAGGTAAGT<br>GGAACTCCATGTGTAGCGGTGGAATGCGTAGATATATGGAAGAACACCAGTGGCGAAG<br>GCGGCTTACTGGACTGCAACTGACGTTGAGGCTCGAAAGTGTGGGTAGCAAACAGGAT<br>TAGATACCCTGGTAGTCCACACCGTAGACGATGAACACTAGGTGTTAGGAGGTTTCCG<br>CCTCTTAGTGACGAAGCTAACGCATTAAGTGTTCCGCCTGGGGAGTACGACCGCAAGG<br>TTGAAACTCAAAGGAATTGACGGGGACCCGCACAAGCGGTGGAGCATGTGCTTTAATT<br>CGAAGCAACGCGAAGAACCTTACCAGGTCTTGACATCCTTTGAAGCTTTTAGAGATAG<br>AGTGTTCTCTTCGGAG	1002	99.5%	100%	*Leuconostoc mesenteroides subsp. dextranicum*	NR_040817.1
294	A1	T0	PCA	GAAGGAGCTTGCTCCTGATTGATAAACATTTGAGTGAGTGGCGGACGGGTGAATAACA<br>CGTGGGTAACCTGCCCTAAAGTGGGGGATAACATTTGGAAACAGATGCTAATACCGCA<br>TAAAAC	122	99.2%	100%	*Latilactobacillus sakei*	NR_113821.1
543	A3	T0	RBCA	AAAAAATCTTTTTGCCGCGCTTAATTGCGCGGCGAAAAGACCTTACACACAGTGTTTTT<br>TGTTATTACAAGAACTTTTGCTTTGGTCTGGACTAGAAATAGTTTGGGCCAGAGGTTTA<br>CTGAACTAAACTTCAATATTTATATTGAATTGTTATTTATTTAATTGTCAATTTGTTGAT<br>TAAATTCAAAAAATCTTCAAAACTTTCAACAACGGATCTCTTGGTTCTCGCATCGATGA<br>AGAACGCAGCGAAATGCGATAAGTAATATGAATTGCAGATTTTCGTGAATCATCGAAT<br>CTTTGAACGCACATTGCGCCCTCTGGTATTCCAGAGGGCATGCCTGTTTGAGCGTCATT<br>TCTCTCTCAAACCTTCGGGTTTGGTATTGAGTGATACTCTTAGTTGAACTAGGCGTTTGC<br>TTGAAATGTATTGGCATGAGTGGTACTGGATAGTGCTATATGACTTTCAATGTATTAGG<br>TTTATCCAACTCGTTGAATAGTTTAATGGTATATTTCTCGGTATTCTAGGCTCGGCCTTA<br>CAATATAACAAACAAGTTTGACCTCAAATCAGGTAGGATTACCCGCTGAACTTAAGCA<br>TATCAATAAGCGGAGGAAGGTAATTACAGTATTCTTTTTGCCAGCGCTTAATTGCGCGG<br>CGAAAAAACCTTACACACAGTGTTTTTTGTTATTACAAGAACTTTTGCTTTGGTCTGGA<br>CTAGAAATAGTTTGGGCCAGAAGTTTACTGAACTAAACTTCAATATTTATATTGAAATG<br>TTATTTATTTAAATGTCAATTGTTGATTAAATTCAAAAACTTCAAACTTTCACAGGGAA<br>C	828	99.7%	98%	*Debaryomyces hansenii*	MH545920.1
464	A1	T12	BPA	GTGCTAATACATGCAAGTCGAGCGAACAGATAAGGAGCTTGCTCCTTTGAAGTTAGCG<br>GCGGACGGGTGAGTAACACGTGGGTAACCTACCTATAAGACTGGGATAACTTCGGGAA<br>ACCGGAGCTAATACCGGATAACATTTAGAACCGCATGGTTCTAAAGTGAAAGATGGTT<br>TTGCTATCACTTATAGATGGACCCGCGCCGTATTAGCTAGTTGGTAAGGTAACGGCTTA<br>CCAGGGCGACGATACGTAGCCGACCTGAGAGGGTGATCGGCCACGCTGGAACTGAGA<br>CACGGTCCAGACTCCTACGGGAGGCAGCAGTAGGGAATCTTCCGCAATGGGCGAAAGC<br>CTGACGGAGCAACGCCGCGTGAGTGATGAAGGGTTTCGGCTCGTAAAACTCTGTTATT<br>AGGGAAGAACAAATGTGTAAGTAACTGTGCACATCTTGACGGTACCTAATCAGAAAGC<br>CACGGCTAACTACGTGCCAGCAGCCGCGGTAATACGTAGGTGGCAAGCGTTATCCGGA<br>ATTATTGGGCGTAAAGCGCGCGTAGGCGGTTTCTTAAGTCTGATGTGAAAGCCCACGG<br>CTCAACCGTGGAGGGTCATTGGAAACTGGGAAACTTGAGTGCAGAAGAGGAAAGTGG<br>AATTCCATGTGTAGCGGTGAAATGCGCAGAGATATGGAGGAACACCAGTGGCGAAGG<br>CGACTTTCTGGTCTGTAACTGACGCTGATGTGCGAAAGCGTGGGGATCAAACAAGGAT<br>TAGATACCCTGGTAGTCCACGCCGTACACGATGAGTGCTAAGTGTTAGGCGGTTTCCGC<br>CCCTTAGTGCTGCAGCTAACGCATTAAGCACTCCGCCTGGGGAGTACGACCGCAAGGT<br>TGAAACTCAGAC	881	99.3%	99%	*Staphylococcus xylosus*	NR_113350.1
466	A1	T12	BPA	GCTTACACATGCAAGTCGAACGCTGAAGCTTGGTGCTTGCACTGGGTGGATGAGTGGC<br>GAACGGGTGAGTAATACGTGAGTAACCTGCCCTTGACTCTGGGATAAGCCTGGGAAAC<br>TGGGTCTAATACTGGATACGACGGCGCATCGCATGGTGTGTTGTGGAAAGGGTTTTACT<br>GGTTTTGGATGGGCTCACGGCCTATCAGCTTGTTGGTGGGGTAATGGCTCACCAAGGCG<br>ACGACGGGTAGCCGGCCTGAAAGGGTGACCGGCCACACTGGGACTGAC	282	96.8%	99%	*Kocuria* sp.	NR_148610.1

Strain ID	Product (tray)	Sample	Medium	Sequence	bp	% identity	Query coverage	Name	Accession n°
385	A1	T12	PCA	ACATGCAGTCGAACGCACAGCGAAAGGTGCTTGCACCTTTCAAGTGAGTGGCGAACGG<br>GTGAGTAACACGTGGACAACCTGCCTCAAGGCTGGGGATAACATTTGGAAACAGATGC<br>TAATACCGAATAAAACTTAGTGTCGCATGACACAAAGTTAAAAGGCGCTTCGGCGTCA<br>CCTAGAGATGGATCCGCGGTGCATTAGTTAGTTGGTGGGGTAAAGGCCTACCAAGACA<br>ATGATGCATAGCCGAGTTGAGAGACTGATCGGCCACATTGGGACTGAGACACGGCCCA<br>AACTCCTACGGGAGGCTGCAGTAGGGAATCTTCCACAATGGGCGAAAGCCTGATGG	346	99.7%	100%	*Leuconostoc mesenteroides subsp. dextranicum*	NR_040817.1
548	A1	T12	RBCA	GAAAGACCTTACACACTATGTTTTTTTGATTTGAAACTTTTGCTTTGGTCTGACTTAGAA<br>ATGAGTTGGGCCAAAGGTTTTATACTAAAACTTCAATTTTATTATTGAATTGTTAATTA<br>ATTATATTGTCAATTGTTGATTAAATTCAAAAATC	154	98.7%	97%	*[Candida] zeylanoides*	NR_131278.1
549	A1	T12	RBCA	AAGACCTTACACACTATGTTTTTTTGATTTGAAACTTTTGCTTTGGTCTGACTTAAAAAT<br>GAGTTGGGCCAAAGGTTTTATACTAAAACTTCATT	95	97.9%	97%	*[Candida] zeylanoides*	NR_131278.1
521	A1	T12	VRBGA	CCATGCAAGTCGAACGGCAGCGCGGGGCAACCTGGCGTGCGAGTGGCGAACGGGTGA<br>GTAATACATCGGAACGTACCCAGAAGTGGGGGATAACGTAGCGAAAGTTACGCTAATA<br>CCGCATACGTTCTACGGAAGAAAGTGGGGGACCTTCGGACCTCATGCTTTTGGAGCGG<br>CCGATGTCTGATTAGCTAGTTGGTGAGGTAAAGGCTCACCAAGGCGACGATCAGTAGC<br>TGGTCTGAGAGGACGACCAGCCACACTGGGACTGAGACACGGCCCAGACTCCTACGGG<br>AGGCAGCAGTGGGGAATTTTGGACAATGGGCGCAAGCCTGATC	332	99.4%	99%	*Massilia arvi*	NR_137346.1
522	A1	T12	VRBGA	CAGCCTAACCACATGACAAGTCGAACGGTAGCACAGAGAGCTTGCTCTTGGGTGACGA<br>GTGGCGGACGGGTGAGTAATGTCTGGGAAACTGCCCGATGGAGGGGGATAACTACTGG<br>AAACGGTGGCTAATACCGCATAACGTCTTCGGACCAAAGTGGGGGACCTTCGGGCCTC<br>TCACCATCGGATGTGCCCAGATGGGATTAGCTAGTAGGTGGGGTAATGGCTCACCTAG<br>GCGACGATCCCTAGCTGGTCTGAGAGGATGACCAGCCACACTGGAACTGAGACACGGT<br>CCAGACTCCTACGGGAGGCAGCAGTGGGGAATATTGCACAATGGGCGCAAGCCTGATG<br>CAGCCATGCCGCGTGTATGAAGAAGGCCTTCGGGTTGTAAAGTACTTTCAGCGGGGAG<br>GAAGGCGATACGGTTAATAACCGTGTCGATTGACGTTACCCGCAGAAAAAGCACCGGC<br>TAACTCCGTGCCAGCAGCCGCGGTAATACGGAGGGTGCAAGCGTTAATCGGAATTACT<br>GGGCGTAAAGCGCACGCAGGCGGTCTGTCAAGTCAGATGTGAAATCCCCGGGCTTAAC<br>CTGGGAACTGCATTTGAAACTGGCAGGCTTGAGTCTCGTAGAGGGGGGTAGAATTCCA<br>GGTGTAGCGGTGAAATGCGTAGAGATCTGGAGGAATACCGGTGGCGAAGGCGGCCCC<br>CTGGACAAAGACTGACGCTCAGGTGCGAGAGCGTGTGGAGCAAACAGGATTAGATAC<br>CCTGGTAGTCCACGCTGTAAACGATGTCGACTTGGAGGTTGTGCCCTTGAGGCGTGGCT<br>TCCGGAGCTAACGCGTTAAGTCGACCGCCTGGGGAGTACGGCCGCAAGGTTAAAACTC<br>TAATGAATTGACGGGGGCGCGCACAAGCGGTGGAGCATGTGGTTTATTTCGAT	922	98.2%	99%	*Erwinia billingiae*	NR_104932.1
16	A2	T0	MRSA	TGCCTAATACATGCAAGTCGAACGCACAGCGAAAGGTGCTTGCACCTTTCAAGTGAGT<br>GGCGAACGGGTGAGTAACACGTGGACAACCTGCCTCAAGGCTGGGGATAACATTTGGA<br>AACAGATGCTAATACCGAATAAAACTTAGTGTCGCATGACACAAAGTTAAAAGGCGCT<br>TCGGCGTCACCTAGAGATGGATCCGCGGTGCATTAGTTAGTTGGTGGGGTAAAGGCCT<br>ACCAAGACAATGATGCATAGCCGAGTTGAGAGACTGATCGGCCACATTGGGACTGAGA<br>CACGGCCCAAACTCCTACGGGAGGCTGCAGTAGGGAATCTTCCACAATGGGCGAAAGC<br>CTGATGGAGCAACGCCGCGTGTGTGATGAAGGCTTTCGGGTCGTAAAGCACTGTTGTA<br>TGGGAAGAACAGCTAGAATAGGAAATGATTTTAGTTTGACGGTACCATACCAGAAAGG<br>GACGGCTAAATACGTGCCAGCAGCCGCGGTAATACGTATGTCCCGAGCGTTATCCGGA<br>TTTATTGGGCGTAAAGCGAGCGCAGACGGTTTATTAAGTCTGATGTGAAAGCCCGGAG<br>CTCAACTCCGGAATGGCATTGGAAACTGGTTAACTTGAGTGCAGTAGAGGTAAGTGGA<br>ACTCCATGTGTAGCGGTGGAATGCGTAGATATATGGAAGAACACCAGTGGCGAAGGCG<br>GCTTACTGGACTGCAACTGACGTTGAGGCTCGAAAGTGTGGGTAGCAAACAGGATTAG<br>ATACCCTGGTAGTCCACACCGTAAACGATGAACACTAGGTGTTAGGAGGTTTCCGCCTC<br>TTAGTGCCGAAGCTAACGCATTAAGTGTTCCGCCTGGGGAGTACGACCGCAAGGTTGA<br>AACTCAAAGGAATTGACGGGGACCCGCACAAGCGGT	907	100.0%	100%	*Leuconostoc mesenteroides subsp. dextranicum*	NR_040817.1
472	A2	T12	BPA	GTGCTAATACTGCAAGTCGAGCGAACAGATAAGGAGCTTGCTCCTTTGAAGTTAGCGG<br>CGGACGGGTGAGTAACACGTGGGTAACCTACCTATAAGACTGGGATAACTTCGGGAAA<br>CCGGAGCTAATACCGGATAACATTTAGAACCGCATGGTTCTAAAGTGAAAGATGGTTT<br>TGCTATCACTTATAGATGGACCCGCGGCGTATTAGCTAGTTGGTAAGGGAGCGGCTTAC<br>CAGGGCGACGATACCTAGCCGGGCTGAGAGGGTGATCGGGCACGCTGGAACTGAGAC<br>ACGGTGCAGGCT	302	96.0%	99%	*Staphylococcus xylosus*	NR_113350.1

Strain ID	Product (tray)	Sample	Medium	Sequence	bp	% identity	Query coverage	Name	Accession n°
178	A2	T12	MRSA	GCGGGGCCTAATACATGCAAGTCGAACGCACAGCGAAAGGTGCTTGCACCTTTCAAGTGAGTGGCGAACGGGTGAGTAACACGTGGACAACCTGCCTCAAGGCTGGGGATAACATTTGGAAACAGATGCTAATACCGAATAAAACTTAGTGTCGCATGACACAAAGTTAAAAGGCGCTTCGGCGTCACCTAGAGATGGATCCGCGGTGCATTAGTTAGTTGGTGGGGTAAAGGCCTACCAAGACAATGATGCATAGCCGAGTTGAGAGACTGATCGGCCACATTGGGACTGAGACACGGCCCAAACTCCTACGGGAGGCTGCAGTAGGGAATCTTCCACAATGGGCGAAAGCCTGATGGAGCAACGCCGCGTGTGTGATGAAGGCTTTCGGGTCGTAAAGCACTGTTGTATGGGAAGAACAGCTAGAATAGGAAATGATTTTAGTTTGACGGTACCATACCAGAAAGGGACGGCTAAATACGTGCCAGCAGCCGCGGTAATGCGTATGTCCCGAGCGTTATCCGGATTTATTGGGCGTAAAGCGAGCGCAGACGGCTTATTAAGTCTGATGTGAAAGCCCGGAGCTCAACTCCGGAATGGCATTGAAAACTGGTTAACTTGAGTGCAGTAGAGG	634	99.2%	100%	*Leuconostoc mesenteroides subsp. dextranicum*	NR_040817.1
551	A2	T12	RBCA	AATTCTATTGCCAGCGCTTAATTGCGCGGCAAAAACCTTACACACTATGTTTTTTTGATTTGAAACTTTTGCTTTGGTCTGACTTAGAAATGAGTTGGGCCAAAGGTTTTATACTAAAACTTCAATTTTATTATTGAATTGTTAATTAATTATATTGTCAATTTGTTGATTAAATTCAAAAATCTTCAAAACTTTCAACAACGGATCTCTTGGTTCTCGCATCGATGAAGAACGCAGCGAAATGCGATAAGTAATATGAATTGCAGATTTTCGTGAATCATCGAATCTTTGAACGCACATTGCGCCCTATGGTATTCCATAGGGCATGCCTGTTTGAGCGTCATTTCTCTCTCAAATCTTCGGATTTGGTTTTGAGTGATACTCTTAGTCAGACTAAGCGTTTGCTTGAAATGTATTGGCATGAGTGGTACTAGATAGTGCTGAACTGTTTCAATGTATTAGGTTTATCCAACTCGTTGACCACTATAGTATTTGTTTATTACACAGGCTCGGCCTTACAACAACAAACAAAGTTTGACCTCAAATCAGGTAGGACTACCCGCTGAACTTAAGCATATCAATAACCGGAGCGAAAGGGAACATTACGGTATTCTTTTGCCGCGCTTAATTGCCCGGCAAAAAACCTTACACACTATGTTTTTTTGATTTGAAACTTTTGCTTTGGGTCGGACTTAAAAATGGAGTTGGGCAAAGGGTTTTATACTAAAACTTCAATTTTATTATTGAAATTGTTAATTAATTATATTGTCAAATTGTTGAATTAACATTCAAAAATCTCAA	801	99.4%	90%	*[Candida] zeylanoides*	NR_131278.1
302	A3	T0	PCA	TGCTATACATGCAGTCGAACGCACAGCGAAAGGTGCTTGCACCTTTCAAGTGAGTGGCGAACGGGTGAGTAACACGTGGACAACCTGCCTCAAGGCTGGGGATAACATTTGGAAACAGATGCTAATACCGAATAAAACTTAGTGTCGCATGACACAAAGTTAAAAGGCGCTTCGGCGTCACCTAGAGATGGATCCGCGGTGCATTAGTTAGTTGGTGGGGTAAAGGCCTACCAAGACAATGATGCATAGCCGAGTTGATAGACTGATCGGCCACTTTGGGACTGA	285	98.9%	98%	*Leuconostoc mesenteroides*	NR_040817.1
477	A3	T12	BPA	ACATGCAAGTCAGAGCGGTAACAGAGGAAAGCTTGCTTTCTTGCTGACAGAGCGGCGGACGGGTGAGTAATGTATGGGGATCTGCCCGATAGAGGGGGATAACTACTGGAAACGGTAGCTAATACCGCATGACGTCTACGGACCAAAGCAGGGGCTCTTCGGACCTTGCGCTATCGGATGAACCCATATGGGATTACCT	199	99.0%	98%	*Proteus hauseri*	NR_104767.1
403	A3	T12	PCA	GCCTAATACATGCAAGTCGAACGCACAGCGAAAGGTGCTTGCACCTTTCAAGTGAGTGGCGAACGGGTGAGTAACACGTGGACAACCTGCCTCAAGGCTGGGGATAACATTTGGAAACAGATGCTAATACCGAATAAAACTTAGTGTCGCATGACACAAAGTTAAAAGGCGCTTCGGCGTCACCTAAAGATGGATCCGCGGTGCATTAGTTAGTTGGTGGGGTAAAGGCCTACCAAGACAATGATGCATAGCCGAGTTGAGAGACTGATCGGCCACATTGGGACTGAGACACGGCCCAAACTCCTACGGGAGGCTGCAGTAGGGAATCTTCCACAACGGGCGAAAGCCTGATGGAGC	357	99.4%	100%	*Leuconostoc mesenteroides subsp. dextranicum*	NR_040817.1
555	A3	T12	RBCA	GGCGAAGACCTTACACACTATGTTTTTTTGATTTAGAAACTTTTGCTTTGGTCTGACTTAGAAATGAGTTGGGCCAAAGGTTTTATACTAAAACTTCAATTTTATT	106	97.2%	100%	*[Candida] zeylanoides*	NR_131278.1
37	B1	T0	MRSA	TGCTAATACTGCAAGTCGAACGCACTCTCGTTTAGATTGAAGGAGCTTGCTCCTGATTGATAAACATTTGAGTGAGTGGCGGACGGGTGAGTAACACGTGGGTAACCTGCCCTAAAGTGGGGGATAACATTTGGAAACAGATGCTAATACCGCATAAAACCTAACACCGCATGGTGTAGGGTTGAAAGATGGTTTCGGCTATCACTTTAGGATGGACCCGCGGTGCATTAGTTAGTTGGTGAGGTAAAGGCTCACCAAGACCGTGATGCATAGCCGACCTGAGAGGGTAATCGGCCACACTGGGACTGAGACACGGCCCAGACTCCTACGGGAGGCAGCAGTAGGGAATCTTCCACAATGGACGAAAGTCTGATGGAGCAACGCCGCGTGAGTGAAGAAGGTTTTCGGATCGTAAAACTCTGTTGTTGGAGAAGAATGTATCTGATAGTAACTGATCAGGTAGTGACGGTATCCAACCAGAAAGCC	486	99.8%	99%	*Latilactobacillus sakei*	NR_113821.1
600	B1	T12	PCA	GCCGATGTCTGATTAGCTAGATGGTGAGGTAAAGGCTCACCAAGGCCACCATCA	54	94.4%	100%	*Massilia aurea*	NR_042502.1

Strain ID	Product (tray)	Sample	Medium	Sequence	bp	% identity	Query coverage	Name	Accession n°
559	B1	T12	RBCA	AAATATCTTTTGCCAGCGCTTAATTGCGCGGCGAAAGACCTTACACACTATGTTTTTTTGATTTGAAACTTTTGCTTTGGTCTGACTTAGAAATGAGTTGGGCCAAAGGTTTTATACTAAAACTTCAATTTTATTATTGAATTGTTAATTAATTATATTGTCAATTTGTTGATTAAATTCAAAAATCTTCAAGACGTTCAACAACGGATCTCTTGGTTCTCGCATCGATGAAGAACGCAGCGAAATGCGATAAGTAATATGAATTGCAGATTTTCGTGAATCATCGAATCTTTGAACGCACATTGCGCCCTATGGTATTCCATAGGGCATGCCTGTTTGAGCGTCATTTCTCTCTCAAATCTTCGGATTTGGTTTTGAGTGATACTCTTAGTCAGACTAAGCGTTTGCTTGAAATGTATTGGCATGAGTGGTACAAGATAGTGCTGAACTGTTACAATGTATG	463	98.9%	98%	*[Candida] zeylanoides*	NR_131278.1
560	B1	T12	RBCA	TTATTCGTTGCTTTGCGGAGGGAGACACTATACTGCTGGACCAGCGCTTAATTGCGCGGTTTGGTGGGTCTCTGTAGCTCAGTAGCACTATTACACACAGTGGAGATTTTTATAATTCTTTGCATGCTTCTTTGGGCTGCTTCGGCGGCCCAGGAGTGACAAACACAAACAATTTTGTAATTTTATGAACTAGTCAAAACCAGAATTCCAGGAAGATTTATCTTTTTGTAATATTAAAACAAATATTAAAACTTTCAACAACGGATCTCTTGGTTCTCGCATCGATGAAGAACGCAGCGAAATGCGATACGTAATGTGAATTGCAGAATTCCGTGAATCATCGAATCTTTGAACGCACATTGCGCCCTCTGGTATTCCAGGGGGCATGCCTGTTTGAGCGTCATTTCCTTCTCAAACAGCAATGTTTGGTTGTGAGTGATACTCTTTCGGAGTTAGCTTGAAATTGCTGGCCGATGGCTGTTGTGGTTGAGTGTCTCCCTTCGGGGAGAGCGCTTGCTGCGTTAAGGGACGTCCTGCTGGACATCTTCGTATTAGGTTCTACCAACTTCGAAGACGGTTAGCGGGGAGTTCTGCAGTGAGTGTAGTGCTTTTACTACGTTGCACCATGGCGAACAGTGTTCTTTTAAGTTTGACCTCAAATCAGGTAGGATTACCCGCTGAACTTAAGCATATCAATACGCGGAGGAAGATC	712	99.5%	91%	*Kazachstania servazzii*	NR_138193.1
562	B1	T12	RBCA	AGAAATATTTTTCGTTGCTTTGCGAGGAGACACTATACTGCTGGACCAGCGCTTAATTGCGCGGTTTGGTGGGTCTCTGTAGCTCAGTAGCACTATTACACACAGTGGAGATTTTTATAATTCTTTGCATGCTTCTTTGGGCTGCTTCGGCGGCCCAGGAGTGACAAACACAAACAATTTTGTAATTTTATGAACTAGTCAAAACCAGAATTCCAGGAAGATTTATCTTTTTGTAATATTAAAACAAATATTAAAACTTTCAACAACGGATCTCTTGGTTCTCGCATCGATGAAGAACGCAGCGAAATGCGATACGTAATGTGAATTGCAGAATTCCGTGAATCATCGAATCTTTGAACGCACATTGCGCCCTCTGGTATTCCAGGGGGCATGCCTGTTTGAGCGTCATTTCCTTCTCAAACAGCAATGTTTGGTTGTGAGTGATACTCTTTCGGAGTTAGCTTGAAATTGCTGGCCGATGGCTGTTGTGGTTGAGTGTCTCCCTTCGGGGAGAGCGCTTGCTGCGTTAAGGGACGTCCTGCTGGACATCTTCGTATTAGGTTCTACCAACTTCGAAGACGGTTAGCGGGGAGTTCTGCAGTGAGTGTAGTGCTTTTACTACGTTGCACCATGGCGAACAGTGTTCTTTTAAGTTTGACCTCAAATCAGGTAGGATTACCCGCTGAACTTAAGCATATCGATA	703	100.0%	92%	*Kazachstania servazzii*	NR_138193.1
545	B2	T0	RBCA	AATTTTTCGTTGCTTTGCGAGGAGACACTATACTGCTGGACCAGCGCTTAATTGCGCGGTTTGGTGGGTCTCTGTAGCTCAGTAGCACTATTACACACAGTGGAGATTTTTATAATTCTTTGCATGCTTCTTTGGGCTGCTTCGGCGGCCCAGGAGTGACAAACACAAACAATTTTGTAATTTTATGAACTAGTCAAAACCAGAATTCCAGGAAGATTTATCTTTTTGTAATATTAAAACAAATATTAAAACTTGCAAC	259	99.6%	100%	*Kazachstania servazzii*	NR_138193.1
202	B2	T12	MRSA	TGCGGCGTGCTAATACATGCAAGTCGAACGCACAGCGAAAGGTGCTTGCACCTTTCAAGTGAGTGGCGAACGGGTGAGTAACACGTGGACAACCTGCCTCAAGGCTGGGGATAACATTTGGAAACAGATGCTAATACCGAATAAAACTTAGTGTCGCATGACACAAAGTTAAAAGGCGCTTCGGCGTCACCTAGAGATGGATCCGCGGTGCATTAGTTAGTTGGTGGGGTAAAGGCCTACCAAGACAATGATGCATAGCCGAGTTGAGAGACTGATCGGCCACATTGGGACTGAGACACGGCCCAAACTCCTACAGGAGGCTGCAGTAAGGAATCTTCCACAGTGGGCGAAAGCCTGATGGATCAACGCCGCGTGTGTGATGAAGGCTTTCGGGT	395	98.7%	99%	*Leuconostoc mesenteroides*	NR_040817.1
523	B2	T12	VRBGA	CCAAAGAGGGGGACCTTCGGGCCTCTCACCATCGGATGTGCCCAGATGGGATTAGCTAGTAGGTGGGGTAATGGCTCACCTAGGCGACAATCCCTAGCTGGTCTGAGAGGATGACCAGCCACACTGGAACTGAGACACGGTCCAGACTCCTACGGGAGGCAGCAGTGGGGAATATTGCACAATGGGCGCAAGCCTGATGCAGCCATGCCGCGTGTATGAAGAAGGCCTTCGGGTTGTAAAGTACTTTCAGCGGGGAGGAAGGCGATACGGTTAATAACCGTGTCGATTGACGTTACCCGCACAAAAAGCACCGGCTAACTCCGTGCCAGCAGCCGCGGTAATACGGAGGGTGCAAGCGTTAATCGGAATTACTGGGCGTAAAGCGCACGCACGCGGTCTGTCAAGTCACATGTGAAATCCCCGGGCTTAACCTGGGAACTGCGTTTGAAACTGGCAGGCTTGAGTCTCGTATAGGGGGGTAGAATTCCAGGTGTAGCGGTGAAAATGCGCACAGATCTGGAGGAATACCGGTGGAGAAG	539	96.9%	100%	*Erwinia billingiae*	NR_104932.1

Strain ID	Product (tray)	Sample	Medium	Sequence	bp	% identity	Query coverage	Name	Accession n°
69	B3	T0	MRSA	CGTGCTAATACATGCAAGTCGAACGCACTCTCGTTTAGATTGAAGGAGCTTGCTCCTGATTGATAAACATTTGAGTGAGTGGCGGACGGGTGAGTAACACGTGGGTAACCTGCCCTAAAGTGGGGGATAACATTTGGAAACAGATGCTAATACCGCATAAAACCTAACACCGCATGGTGTAGGGTTGAAAGATGGTTTCGGCTATCACTTTAGGATGGACCCGCGGTGCATTAGTTAGTTGGTGAGGTAAAGGCTCACCAAGACCGTGATGCATAGCCGACCTGAGAGGGTAATCGGCCACACTGGGACTGAGACACGGCCCAGACTCCTACGGGAGGCAGCAGTAGGGAATCTTCCACAATGGACGAAAGTCTGATGGAGCAACGCCGCGTGAGTGAAGAAGGTTTTCGGATCGTGAAACTCTGTTGTTGGAGAATAATGTATCTGATAGTAACTGATCAGGTAGTGACGGTATC	476	99.4%	100%	*Latilactobacillus sakei*	NR_113821.1
420	B3	T12	PCA	GCTATACATGCAAGTCGAACGCACTCTCGTTTAGATTGAAGGAGCTTGCTCCTGATTGATAAACATTTGAGTGAGTGGCGGACGGGTGAGTAACACGTGGGTAACCTGCCCTAAAGTGGGGGATAACATTTGGAAACAGATGCTAATACCGCATAAAACCTAACACCGCATGGTGTAGGGTTGAAAGATGGTTTCGGCTATCACTTTAGGATGGACCCGCGGTGCATTAGTTAGTTGGTGAGGTAAAGGCTCACCAAGACCGTGATGCATAGCCGACCTGAGAGGGTAATCGGCCACACTGGGACTGAGACACGGGCCAAACTCCTACGGGAGGCAGC	338	99.4%	99%	*Latilactobacillus sakei*	NR_113821.1
332	C1	T0	PCA	TGCTATACATGCAAGTCGAACGCACAGAAGTTAGAAGAGCTTGCTCTTTAACCAAGTGAGTGGCGGACGGGTGAGTAACACGTGGGTAACCTGCCCATTAGAGGGGGATAACATTCGGAAACGGATGCTAATACCGCATAGTTTCAGGAATCGCATGATTCTTGAAGGAAAGGTGGCTTCGGCTACCACTAATGGATGGACCCGCGGCGTATTAGCTAGTTGGTGAGGTAATGGCTCACCAAGGCAATGATACGTAGCCGACCTGAGAGGGTGATCGGCCACACTGGGACTGAGACACGGCCCAGACTCCTACGGGA	317	97.8%	98%	*Carnobacterium gallinarum*	NR_042093.1
423	C1	T12	PCA	ACGGCGGCGTGCTAATACATGCAAGTCGAACGCACAGCGAAAGGTGCTTGCACCTTTCAAGTGAGTGGCGAACGGGTGAGTAACACGTGGACAACCTGCCTCAAGGCTGGGGATAACATTTGGAAACAGATGCTAATACCGAATAAAACTTAGTATCGCATGATACCAGGTTAAAAGGCGCTACGGCGTCACCTAGAGATGGATCCGCGGTGCATTAGTTAGTTGGTGGGGTAAAGGCCTACCAAGACAATGATGCATAGCCGAGTTGAGAGACTGATCGGCCACATTGGGACTGAGACACGGCCCAAACTCCTACGGGAGGCTGCAGTATGGAATCTTCCACAATGGGCGAAAGCCTGATGGAGCAACGCCGCGTGTGTGATGAAGGCTTTCGGGTCGTAA	402	99.5%	99%	*Leuconostoc carnosum*	NR_040811.1
425	C1	T12	PCA	CCTATACATGCAAGTCGAACGCACGAAGT	29	100.0%	98%	*Carnobacterium* sp.	NR_044710.2
225	C2	T12	MRSA	CGTGCCTAATACATGCAAGTCGAACGCACAGCGAAAGGTGCTTGCACCTTTCAAGTGAGTGGCGAACGGGTGAGTAACACGTGGACAACCTGCCTCAAGGCTGGGGATAACATTTGGAAACAGATGCTAATACCGAATAAAACTTAGTATCGCATGATACCAGGTTAAAAGGCGCCACG	179	99.4%	100%	*Leuconostoc carnosum*	NR_040811.1
494	C3	T12	BPA	CCATGCAAGTCAGAACGGAAAGGCCTTGTGCTTGCACAAGGTACTCGAGTGGCGAACGGGTGAGTAACACGTGGGTGATCTGCCCTGCACTGTGGGATAAGCCTGGGAAACTGGGTCTAATACCATATAGGACCGCATCTTGGATGGTGTGGTGGAAAGCTTTTGCGGTGTGGGATGAGCCTGCGGCCTATCACCTTGTTGGTGGGGTAATGGCCTACCAAGGCGGCGACGGGTATCCGGCCTGAGAGGGT	251	99.2%	99%	*Corynebacterium stationis*	NR_116558.1
230	C3	T12	MRSA	ATACATGCAAGTCGAACGCACAGCGAAAGGTGCTTGCACCTTTCAAGTGAGTGGCGAACGGGTGAGTAACACGTGGACAACCTGCCTCAAGGCTGGGGATAACATTTGGAAACAGATGCTAATACCGAATAAAACTTAGTATCGCATGATACCAGGTTAAAAGG	164	100.0%	100%	*Leuconostoc carnosum*	NR_040811.1
431	C3	T12	PCA	ATGCAGTCGAACGCACAGCAGAAAGGTGCTTGCACCTTTCAAGTGAGTGGCGAACGGGTGAGTAACACGTGGACAACCTGCCTCAAGGCTGGGGATAACATTTGGAAACAGATGCTAATACCGAATAAAACTTAGTATCGCATGATACCAGGTTAAA	157	98.7%	100%	*Leuconostoc carnosum*	NR_040811.1
112	D1	T0	MRSA	GAAGTTGAAAAGCTTGCTTTTCGACCAAGTGAGTGGCGGACGGGTGAGTAACACGTGGGTAACCTGCCCATAAGAGGGGGATAACATCCGGAAACGGATGCTAATACCGCATATTTCAAATGACCGCATGGTCGCTTGATGAAAGGTGGCTTCGGCTACCGCTTATGGATGGACCCGCGGTGCATTAGTTAGTTGGTGAGGTAATGGCTCACCAAGACAATGATGCATAGCCGACCTGAGAGG	243	99.4%	100%	*Carnobacterium divergens*	NR_113798.1
340	D1	T0	PCA	TAACATCCGGAAACGGATGCTAATACCGCATATTTCAAATGACCGCATGGTCGCTTGATGAAAGGTGGCTTCGGATAC	78	96.2%	100%	*Carnobacterium divergens*	NR_044706.2

Strain ID	Product (tray)	Sample	Medium	Sequence	bp	% identity	Query coverage	Name	Accession n°
351	D1	T0	PCA	CGGCGGCAGGCCTAACACATGCAAGTCGAGCGGTAACAGAAGAAAGCTTGCTTTCTTG<br>CTGACGAGCGGCGGACGGGTGAGTAATGTATGGGGATCTGCCCGATAGAGGGGGATA<br>ACTACTGGAAACGGTAGCTAATACCGCATGACGTCTACGGACCAAAGCAGGGGCTCTT<br>CGGACCTTGCGCTATCGGATGAACCCATATGGGATTAGCTAGTAGGTGAGGTAATGGC<br>TCACCTAGGCGACGATCTCTAGCTGGTCTGAGAGGATGATCAGCCACACTGGGACTGA<br>GACACGGCCCAGACTCCTACGGGAGGCAGCAGTGGGGAATATTGCACAATGGGCGCA<br>AGCCTGATGCAGCCATGCCGCGTGTATGAAGAAGGCCTTAGGGTTGTAAAGTACTTTC<br>AGCGGGGAGGAAGGTGTTAAGATTAATACTCTTAGCAATTGACGTTACCCGCAGAAGA<br>AGCACCGGCTAACTCCGTGCCAGCAGCCGCGGTAATACGGAGGGTGCAAGCGTTAATC<br>GGAATTACTGGGCGTAAAGCGCACGCAGGCGGTCAATTAAGTCAGATGTGAAAGCCCC<br>GAGCTTAACTTGGGAATTGCATCTGAAACTGGTTGGCTAGAGTCTTGTAGAGGGGGGT<br>AGAATTCCACGTGTAGCGGTGAAATGCGTAGAGATGTGGAGGAATACCGGTGGCGAA<br>GGCGGCCCCCTGGACAAAGACTGACGCTCAGGTGCGAAAGCGTGGGGAGCAAACAGG<br>ATTAGATACCCTGGTAGTCCACGCTGTAAACGATGTCGATTTGGAGGTTGTGCCCTTGA<br>GGCGTGGCTTCCGGAGCTAACGCGTTAAATCGACCGCCTGGGGAGTACGGCCGCAAGG<br>TTAAAACTCAGATGAATTGACGGGGGCCCGCACAGCGGTGGAGCATGTGGTTAATTCG<br>ATGCATCGCGAGAACCTTACCTACTCTTGACATCCAAAGAATCCTTTAGAGATAGAGG<br>AGTGCCTTCGGGAACTCTGAAAAGG	1008	99.3%	99%	*Proteus hauseri*	NR_1047<br>67.1
497	D1	T0	VRBGA	GGCGGCGGCCTAACACATGCAAGTCGAGCGGTAACAGAAGAAAGCTTGCTTTCTTGCT<br>GACGAGCGGCGGACGGGTGAGTAATGTATGGGGATCTGCCCGATAGAGGGGGATAAC<br>TACTGGAAACGGTAGCTAATACCGCATGACGTCTACGGACCAAAGCAGGGGCTCTTCG<br>GACCTTGCGCTATCGGATGAACCCATATGGGATTAGCTAGTAGGTGAGGTAATGGCTC<br>ACCTAGGCGACGATCTCTAGCTGGTCTGAGAGGATGATCAGCCACACTGGGACTGAGA<br>CACGGCCCAGACTCCTACGGGAGGCAGCAGTGGGGAATATTGCACAATGGGCGCAAG<br>CCTGATGCAGCCATGCCGCGTGTATGAAGAAGGCCTTAGGGTTGTAAAGTACTTTCAG<br>CGGGGAGGAAGGTGTTAAGATTAATACTCTTAGCAATTGACGTTACCCGCAGAAGAAG<br>CACCGGCTAACTCCGTGCCAGCAGCCGCGGTAATACGGAGGGTGCAAGCGTTAATCGG<br>AATTACTGGGCGTAAAGCGCACGCAGGCGGTCAATTAAGTCAGATGTGAAAGCCCCGA<br>GCTTAACTTGGGAATTGCATCTGAAACTGGTTGGCTAGAGTCTTGTAGAGGGGGGTAG<br>AATTCCACGTGTAGCGGTGAAATGCGTAGAGATGTGGAGGAATACCGGTGGCGAAGGC<br>GGCCCCCTGGACAAAGACTGACGCTCAGGTGCGAAAGCGTGGGGAGCAAACAGGATT<br>AGATACCCTGGTAGTCCACGCTGTAAACGATGTCGATTTGGAGGTTGTGCCCTTGAGGC<br>GTGGCTTCCGGAGCTAACGCGTTAAATCGACCGCCTGGGGAGTACGGCCGCAAGGTTA<br>AAACTCAAATGAATTGACGGGGGCCCGCACAAGCGGTGGAGCATGTGGTTTAATTCGA<br>TGCAACGCGAAGAACCTTAGCTACTCTTGACATCCAGAGAATCCTTTAGAGATAGAGG<br>AGTGCCTTCGGGAACTCTGAGACAGGTGCTGCATGGCTGTCGTCAGCTCGTGTTGTGAA<br>ATGATGG	1050	99.6%	100%	*Proteus hauseri*	NR_1047<br>67.1
501	D1	T0	VRBGA	GGGCGGCAGGCCTAACACATGCAAGTCGAGCGGTAACAGGAAGAAGCTTGCTTCTTTG<br>CTGACGAGCGGCGGACGGGTGAGTAATGTATGGGGATCTGCCTGACAGAGGGGGATA<br>ACTACTGGAAACGGTAGCTAATACCGCATAATCTCTAAGGAGCAAAGCAGGGGACCTT<br>CGGGCCTTGCGCTGTCGGATGAACCCATATGGGATTAGCTAGTAGGTGAGGTAATGGC<br>TCACCTAGGCGACGATCTCTAGCTGGTCTGAGAGGATGATCAGCCACACTGGGACTGA<br>GACACGGCCCAGACTCCTACGGGAGGCAGCAGTGGGGAATATTGCACAATGGGCGCA<br>AGCCTGATGCAGCCATGCCGCGTGTATGAAGAAGGCCTTATGGTTGTAAAGTACTTTCA<br>GTCGGGAGGAAGGCGTTGATATTAATAATATCAGCGATTGACGTTACCGACAGAAGAA<br>GCACCGGCTAACTCCGTGCCAGCAGCCGCGGTAATACGGGGGGTGCAAGCGTTAATCG<br>GAATTACTGGGCGTAAAGCGCACGCAGGCGGTTGATTAAGTTAGATGTGAAATCCCCG<br>GGCTTAACCTGCGAATGGCATCTAAAACTGGTCAGCTAGAGTCTTGTAAA	629	99.2%	99%	*Moellerella wisconsensis*	NR_1049<br>39.1

Strain ID	Product (tray)	Sample	Medium	Sequence	bp	% identity	Query coverage	Name	Accession n°
478	D1	T12	BPA	GGCGGCAGGCCTAACACATGCAAGTCGAGCGGTAACAGAGGAAAGCTTGCTTTCTTGC<br>TGACGAGCGGCGGACGGGTGAGTAATGTATGGGGATCTGCCCGATAGAGGGGGATAA<br>CTACTGGAAACGGTAGCTAATACCGCATGACGTCTACGGACCAAAGCAGGGGCTCTTC<br>GGACCTTGCGCTATCGGATGAACCCATATGGGATTAGCTAGTAGGTGAGGTAATGGCT<br>CACCTAGGCGACGATCTCTAGCTGGTCTGAGAGGATGATCAGCCACACTGGGACTGAG<br>ACACGGCCCAGACTCCTACGGGAGGCAGCAGTGGGGAATATTGCACAATGGGCGCAA<br>GCCTGATGCAGCCATGCCGCGTGTATGAAGAAGGCCTTAGGGTTGTAAAGTACTTTCA<br>GCGGGGAGGAAGGTGTTAAGATTAATACTCTTAGCAATTGACGTTACCCGCAGAAGAA<br>GCACCGGCTAACTCCGTGCCAGCAGCCGCGGTAATACGGAGGGTGCAAGCGTTAATCG<br>GAATTACTGGGCGTAAAGCGCACGCAGGCGGTCAATTAAGTCAGATGTGAAAGCCCCG<br>AGCTTAACTTGGGAATTGCATCTGAAACTGGTTGGCTAGAGTCTTGTAGAGGGGGGTA<br>GAATTCCACGTGTAGCGGTGAAATGCGTACAGATGTGGAGGAATACCGGTGGCGA	691	99.9%	100%	*Proteus hauseri*	NR_104767.1
243	D1	T12	MRSA	GGCGGCGTGCTAATACATGCAAGTCGAACGCTTTGTGGTCCAACTGATTTGAAGAGCTT<br>GCTCAGATATGACGATGGACATTGCAAAGAGTGGCGAACGGGTGAGTAACACGTGGG<br>AAACCTACCTCTTAGCAGGGGATAACACTTGGAAACAAGTGCTAATACCGTATAACAC<br>TAATAACCGCATGGTTATTAGTTAAAAGATGGTCTTGCTATCACTAAGAGATGGTCCCG<br>CGGTGTATTAGCTAGTTGGTAAGGTAATGGCTTACCAAGGCAATGATACATAGCCGAG<br>TTGAGAGACTGATCGGCCACAATGGGACTGAGACACGGCCCATACTCCTACGGGAGGC<br>AGCAGTAGGGAATCTTCCACAATGGACGAAAGTCTGATGGAGCAACGCCGCGTGTGTG<br>ATGAAGGGTTTCGGGTCGTAAAACACTGTTGTAAGAGAAGAATGACATTGAGAGTAAC<br>TGTTCAGTGTGTGACGGTATCTTACCAGAAAGGAACGGCTAAATAGATGCCAGCAGCC<br>GCGGTAATA	532	99.3%	100%	*Weissella viridescens*	NR_040813.1
569	D1	T12	RBCA	CCTTACACAACGTGTTTTTTTATTATAGAACTATTACTTTGGTTTGGCTAAGAAATTAGT<br>TGAGCCAGAGGTGATTTAAACTTCAATTTTATTGAATTGTTATTTTAATTTTTGTCAATT<br>TGTTGATTAAATTCAAAACAATCTTCAAAACTTTCAACAACGGATCTCTTGGTTCTCGC<br>ATCGATGAAGAACGCAGCGAAATGCGATAAGTAATATGAATTGCAGATTTTCGTGAAT<br>CATCGAATCTTTGAACG	254	99.2%	100%	*[Candida] glaebosa*	NR_077183.1
352	D2	T0	PCA	CGAAGTTGAAGAGCTTGCTCTTTAACCAAGTGAGTGGCGGACGGGTGAGTAACACGTG<br>GGTAACCTGCCCATTAGAGGGGGATAACATTCGGAAACGGATGCTAATA	107	94.4%	100%	*Carnobacterium maltaromaticum*	NR_044710.2
570	D2	T12	RBCA	GGAAGTTCTGCGCAGCGATGCGCGGGACTTTCCTTCTACCCTTGTGTATTTATGCGAGA<br>TTGCTTTGGCTAATTTCTAGCCGGAGACTACAACCAAACTAATTTTTTTACAATGCTGTC<br>ACACAACAACATAATATTTAAAACTTTCAACAACGGATCTCTTGGTTCTCGCAACGATG<br>AAGAACGCAGCGAATTGCGATACGTATTGTGAATTGCAGATTTTCGTGAGTCATCGAA<br>TCTTTGAACGCACATTGCACCCTGTGGTATTCCACAGGGTATGCCTGTTTGAGCGTCAT<br>TTCCTTCTCAAGCGTGAGCTTGGTGTTGGCGGAGGTCTTTCGAGGCCCCGCTGAAATAC<br>GCAGGGGGTGCGTGGAAACGAGCTTTCTCTCTACTAATGTCTAGGTTCTGCCAACTCAT<br>TGGACGAGCGTCTGCTGGCTCCACAATCCCACCCCCCCACTAACACACAAACATTGAC<br>CTCAAATCAGGTAGGACTACCCGCTGAAGTTAAGCATATCAATAAGCGGGAGGAAAAA<br>GATCATTATTATTGGAGTTCTGCGCAGCGATGCGGGGGACTTTCTTCTACCTTGTGTATT<br>TATGCGAGATTGCTTTGGCTAATTTCTAGCGGAGACTAA	628	97.8%	92%	*[Candida] norvegica*	NR_111209.1
441	D3	T12	PCA	GCGGTTTGCTTTGGTCTGACTTAGAAATGAGTTGGGCCAGAGGTTTTATACTAAAACTT<br>CAATTTTATTATTGAATTGTTAATTAATTATATTGTCAATTTGTTGATTAAATTCAAAAA<br>TCTTCAAAACTTTCAACAACGGATCTCTTGGTTCTCGCATCGATGAAAAACGCAGCAAA<br>ATGCTATAATTAATATGAATTGCAGATTTTCGTGAATCATCGAATCTTTGAACGCACAT<br>TGCGCCCTATGGTATTCCATACGTCATGCCTGTTTGAGCGTCATTTCTCTCTCACATCTT<br>CGGACTTG	305	97.0%	98%	*[Candida] zeylanoides*	NR_131278.1
445	D3	T12	PCA	CAGTCAGAACGCACAGAAGTTGAAAAGCTTGCTTTTCGACCAAGTGAGTGGCGGACGG<br>GTGAGTAACACGTGGGTAACCTGCCCATAAGAGGGGGATAACATTCGGAAACGGATGC<br>TAATACCGCATATTTCAAGTGACCGCATGGTCGCTTGATGAAAGGTGGCTTCGGCTACC<br>ACTTATGGATGGACCCGCGGTGCATTAGTTAGTTGGTGAGGTAATGGCTCACCAAGAC<br>CATGATGCATAGCCGACCTGAGAGGGTGATCGGCCACACTGGGACTGAGACACGGCCC<br>AGACTCCTACGGGAGGCAGC	311	99.4%	99%	*Carnobacterium divergens*	NR_113798.1

Strain ID	Product (tray)	Sample	Medium	Sequence	bp	% identity	Query coverage	Name	Accession n°
365	E1	T0	PCA	TGCGGCATGCCTAATACATGCAAGTCGAACGCACGAAGTTGAAAAGCTTGCTTTTCGA<br>CCAAGTGAGTGGCGGACGGGTGAGTAACACGTGGGTAACCTGCCCATAAGAGGGGGA<br>TAACATTCGGAAACGGATGCTAATACCGCATATTTCAAGTGACCGCATGGTCGCTTGAT<br>GAAAGGTGGCTTCGGCTACCACTTATGGATGGACCCGCGGTGCATTAGTTAGTTGGTG<br>AGGTAATGGCTCACCAAGACCATGATGCATAGCCGACCTGAGAGGGTGATCGGCCACA<br>CTGGGACTGAGACACGGCCCAGACTCCTACGGGAGGCAGCAGTAGGGAATCTTCCGCA<br>ATGGACGAAAGTCTGACGGAGCAATGCCGCGTGAGTGAAGAAGGTTTTCGGAGCGTAA<br>AACTCTGTTGTTAGAGAAGAACAAGGATGAGAGTAACTGCTCATCCCCTGACGGTATC<br>TAACCGGA	472	99.8%	99%	*Carnobacterium divergens*	NR_113798.1
490	E1	T12	BPA	CGGCGGCAGGCCTAACACATGCAAGTCGAGCGGTAACAGAGGAAAGCTTGCTTTCTTG<br>CTGACGAGCGGCGGACGGGTGAGTAATGTATGGGGATCTGCCCGATAGAGGGGGATA<br>ACTACTGGAAACGGTAGCTAATACCGCATGACGTCTACGGACCAAAGCAGGGGCTCTT<br>CGGACCTTGCGCTATCGGATGAACCCATATGGGATTAGCTAGTAGGTGAGGTAATGGC<br>TCACCTAGGCGACGATCTCTAGCTGGTCTGAGAGGATGATCAGCCACACTGGGACTGA<br>GACACGGCCCAGACTCCTACGGGAGGCAGCAGTGGGGAATATTGCACAATGGGCGCA<br>AGCCTGATGCAGCCATGCCGCGTGTATGAAGAAGGCCTTAGGGTTGTAAAGTACTTTC<br>AGCGGGGAGGAAGGTGTTAAGATTAATACTCTTGGCAATTGACGTTACCCGCAGAAGA<br>AGCACCGGCTAACTCCGTGCCAGCAGCCGCGGTAATACGGAGGGTGCAAGCGTTAATC<br>GGAATTACTGGGCGTAAAGCGCACGCAGGCGGTCAATTAAGTCAGATGTGAAAGCCCC<br>GAGCTTAACTTGGGAATTGCATCTGAAACTGGTTGGCTAGAGTCTTGTAGAGGGGGGT<br>AGAATTCCACGTGTAGCGGTGAAATGCGTAGAGATGTGGAGGAATACCGGTGGCGAA<br>GGCGGCCCCCTGGACAAAGACTGACGCTCAAGTGCGAAAGCGTGCGGAGCAAACAGG<br>ATTAGATACCCTGGTAGTCCACGCTGTAAACGATGTCGATTTGGAGGTTGTGCCCTTGA<br>GGCGTGGCTTCCGGAGCTAACGCGTTAAATCGACCGCCTGAGGAGTACGGCCGCAAGG<br>TTAAAACTCAAATGAATTGACAGGG	892	99.4%	99%	*Proteus hauseri*	NR_104767.1
491	E1	T12	BPA	CGGCGGCAGGCCTAACACATGCAAGTCGAGCGGTAACAGAGGAAAGCTTGCTTTCTTG<br>CTGACGAGCGGCGGACGGGTGAGTAATGTATGGGGATCTGCCCGATAGAGGGGGATA<br>ACTACTGGAAACGGTAGCTAATACCGCATGACGTCTACGGACCAAAGCAGGGGCTCTT<br>CGGACCTTGCGCTATCGGATGAACCCATATGGGATTAGCTAGTAGGTGAGGTAATGGC<br>TCACCTAGGCGACGATCTCTAGCTGGTCTGAGAGGATGATCAGCCACACTGGGACTGA<br>GACACGGCCCAGACTCCTACGGGAGGCAGCAGTGGGGAATATTGCACAATGGGCGCA<br>AGCCTGATGCAGCCATGCCGCGTGTATGAAGAAGGCCTTAGGGTTGTAAAGTACTTTC<br>AGCGGGGAGGAAGGTGTTAAGATTAATACTCTTGGCAATTGACGTTACCCGCAGAAGA<br>AGCACCGGCTAACTCCGTGCCAGCAGCCGCGGTAATACGGAGGGTGCAAGCGTTAATC<br>GGAATTACTGGGCGTAAAGCGCACGCAGGCGGTCAATTAAGTCAAATGTGAAAGCCCC<br>GAGCTTAACTTGCGAATTGCATCTGAAACTGGTTG	613	99.5%	99%	*Proteus hauseri*	NR_104767.1
493	E1	T12	BPA	CGGCGGCAGGCCTAACCACATGCAAGTCGAGCGGTAACAGAGGAAAGCTTGCTTTCTT<br>GCTGACGAGCGGCGGACGGGTGAGTAATGTATGGGGATCTGCCCGATAGAGGGGGAT<br>AACTACTGGAAACGGTAGCTAATACCGCATGACGTCTACGGACCAAAGCAGGGGCTCT<br>TCGGACCTTGCGCTATCGGATGAACCCATATGGGATTAGCTAGTAGGTGAGGTAATGG<br>CTCACCTAGGCGACGATCTCTAGCTGGTCTGAGAGGATGATCAGCCACACTGGGACTG<br>AGACACGGCCCAGACTCCTACGGGAGGCAGCAGTGGGGAATATTGCACAATGGGCGC<br>AAGCCTGATGCAGCCATGCCGCGTGTATGAACAATGCCTGAGGGTTGTAAAGCACTTG<br>CAGCGTGGA	413	98.3%	99%	*Proteus hauseri*	NR_104767.1

Strain ID	Product (tray)	Sample	Medium	Sequence	bp	% identity	Query coverage	Name	Accession n°
282	E1	T12	MRSA	GTGCCTAATACATGACAAGTCGAACGCACTCTCGTTTAGATTGAAGGAGCTTGCTCCTG<br>ATTGATAAACATTTGAGTGAGTGGCGGACGGGTGAGTAACACGTGGGTAACCTGCCCT<br>AAAGTGGGGGATAACATTTGGAAACAGATGCTAATACCGCATAAAACCTAACACCGCA<br>TGGTGTAGGGTTGAAAGATGGTTTCGGCTATCACTTTAGGATGGACCCGCGGTGCATTA<br>GTTAGTTGGTGAGGTAAAGGCTCACCAAGACCGTGATGCATAGCCGACCTGAGAGGGT<br>AATCGGCCACACTGGGACTGAGACACGGCCCAGACTCCTACGGGAGGCAGCAGTAGG<br>GAATCTTCCACAATGGACGAAAGTCTGATGGAGCAACGCCGCGTGAGTGAAGAAGGTT<br>TTCGGATCGTAAAACTCTGTTGTTGGAGAAGAATGTATCTGATAGTAACTGATCAGGTA<br>GTGACGGTATCCAACCAGAAAGCCACGGCTAACTACGTGCCAGCAGCCGCGGTAATAC<br>GTAGGTGGCAAGCGTTGTCCGGATTTATTGGGCGTAAAGCGAGCGCAGGCGGTTTCTT<br>AAGTCTGATGTGAAAGCCTTCGGCTCAACCGAAGAAGTGCATCGGAAACTGGGAAACT<br>TGAGTGCAGAAGAGGACAGTGGAACTCCATGTGTAGCGGTGAAATGCGTAGATATATG<br>GAAGAACACCAGTGGCGAAGGCGGCTGTCTGGTCTGTAACTGACGCTGAGGCTCGAAA<br>GCATGGGTAGCAAACAGGATTAGATACCCTGGTAGTCCATGCCGTAAACGATGAGTGC<br>TAGGTGTTGGAGGGTTTCCGCCCTTCAGTGCCGCAGCTAACGCATTAAGCACTCCGCCT<br>GGGGAGTACGACCGCAAGGTTGAAACTCAAAGGAATTGACGGGGGCCCGCACAAGCG<br>GTGGAGCATGTGGTTTAATTCGAAGCAACGCGAAGAACCTTACCAGGTCTTGACATCC<br>TTTGACCACTCTAGAGATAGAGCTTTCCCTTCGGGGACAAAGTGACAGGTGGTGCATG<br>GTTGTCGTCAGCTCGTGTCGTGAGATGTTGGGTTAAGTCCCGCAACGAGCGCAACCCTT<br>ATTACTAGTTGCCAGCATTTAGTTGGGA	1134	99.8%	99%	*Latilactobacillus sakei*	NR_113821.1
283	E1	T12	MRSA	CTGCTAATACATGCAAGTCGAACGCACTCTCGTTTAGATTGAAGGAGCTTGCTCCTGAT<br>TGATAAACATTTGAGTGAGTGGCGGACGGGTGAGTAACACGTGGGTAACCTGCCCTAA<br>AGTGGGGGATAACATTTGGAAACAGATGCTAATACCGCATAAAACCTAACACCGCATG<br>GTGTAGGGTTGAAAGATGGTTTCGGCTATCACTTTAGGATGGACCCGCGGTGCATTAGT<br>TAGTTGGTGAGGTAAAGGCTCACCAAGACCGTGATGCATAGCCGACCTGAGAGGGTAA<br>TCGGCCACACTGGGACTGAGACACGGCCCAGACTCCTACGGGAGGCAGCAGTAGGGA<br>ATCTTCCACAATGGACGAAAGTCTGATGGAGCAACGCCGCGTGAGTGAAGAAGGTTTT<br>CGGATCGTAAAACTCTGTTGTTGGAGAAGAATGTATCTGATAGTAACTGATCAGGTAG<br>TGACGGTATCCAACCAGAAAGCCACGGCTAACTACGTGCCAGCAGCCGCGGTAATACG<br>TAGGTGGCAAGCGTTGTCCGGATTTATTGGGCGTAAAGCGAGCGCAGGCGGTTTCTTA<br>AGTCTGATGTGAAAGCCTTCGGCTCAACCGAAGAAGTGCATCGGAAACTGGGAAACTT<br>GAGTGCAGAAGAGGACAGTGGAACTCCATGTGTAGCGGTGAAATGCGTAGATATATGG<br>AAGAACACCAGTGGCGAAGGCGGCTGTCTGGTCTGTAACTGACGCTGAGGCTCGAAAG<br>CATGGGTAGCAAACAGGATTAGATGCCCTGGTAGACCATGCCGTATACGATGAGTGCT<br>AGGTGTTGGAGGGTTTCCGCCCTTCAGTGCCGCAGCTAACGCATTAAGCACTCCG	868	99.7%	99%	*Latilactobacillus sakei*	NR_113821.1
449	E1	T12	PCA	GCGGCATGCCTAATACATGCAAGTCGAACGCACGAAGTTGAAAAGCTTGCTTTTCGAC<br>CAAGTGAGTGGCGGACGGGTGAGTAACACGTGGGTAACCTGCCCATAAGAGGGGGAT<br>AACATCCGGAAACGGATGCTAATACCGCATATTTCAAATGACCGCATGGTCGCTTGAT<br>GAAAGGTGGCTTCGGCTACCGCTTATGGATGGACCCGCGGTGCATTAGTTAGTTGGTG<br>AGGTAATGGCTCACCAAGACAATGATGCATAGCCGACCTGAGAGGGTGATCGGCCACA<br>CTGGGACTGAGACACGGCCCAGACTCCTACGGGAGGCAGCAGTAGGGAATCTTCCGCA<br>ATGGACGAAAGTCTGACGGAGCAATGCCGCGTGAGTGAAGTAAGGTTTTCGGATCGTG<br>CAACTCTGTTGTTAGAGAAGAACGAT	431	98.4%	99%	*Carnobacterium divergens*	NR_113798.1
577	E1	T12	RBCA	GGAAGTTGCTTAATTGCATCACACATGTTTTTTTAGAGAACTTGCTTGCAAGAACACTA<br>ATAATTTACTTAGTCAACCAATATAAATATCAAAACTTTCAACAACGGATCTCTTGGTT<br>CTCGCATCGATGAAGAACGCAGCGAAATGCGATACGTAATATGAATTGCAGATTTTCG<br>TGAATCATCGAATCTTTGAACGCACATTGCGCCCTGTGGTATTCCACAGGGCATGCCTG<br>TTTGAGCGTCATTTCTCCCTCAAACCTCTGGTTTGGCGTTGAGTGATACTCGGTTTACTT<br>GAAATACATGAAAAGCATAACTATTAGGTTTTACCAACTCGTTATACTAATCTACAAGT<br>TTGACCTCAAATCAGGTAGGACTACCCGCTGAACTTAAGCATATCAATAAGCAAGA	410	98.5%	97%	*[Candida] sake*	NR_151807.1
288	E2	T12	MRSA	CTGCTAATACTGCAAGTCGAACGCACTCTCGTTAGATTGAAGAAGCTTGCTTCTGATTG<br>ATAACATTTGAGTGAGTGGCGGACGGGTGAGTAACACGTGGGTAACCTGCCCTAAAGT<br>GGGGGATAACATTTGGAAACAGATGCTAATACCGCATAAAACCTAGCACCGCATGGTG<br>CAAGGTTGAAAGATGGTTTCGGCTATCACTTTAGGATGGACCCGCGGTGCATTAGTTAG<br>TTGGTGAGGTAAAGGCTCACCAAGACCGTGATGCATAGCCGACCTGAGAGGGTAATCG<br>GCCACACTGGGACTGAGACACGGCCCAGACTCCTACGGGAGGCAGCAGTAGGGAATCT<br>TCCACAATGGACGAAAGTCTGATGGAGCAACGCCGCGTGAGTGAAGAAGGTTTTCGGA<br>TCGTAAAACTCTGTTGTTGGACAAGAAG	436	99.3%	99%	*Latilactobacillus curvatus*	NR_113334.1

Strain ID	Product (tray)	Sample	Medium	Sequence	bp	% identity	Query coverage	Name	Accession n°
455	E2	T12	PCA	GCGGCATGCCTAATACATGCAAGTCGAACGCACGAAGTTGAAAAGCTTGCTTTTCGAC<br>CAAGTGAGTGGCGGACGGGTGAGTAACACGTGGGTAACCTGCCCATAAGAGGGGGAT<br>AACATTCGGAAACGGATGCTAATACCGCATATTTCAAGTGACCGCATGGTCGCTTGAT<br>GAAAGGTGGCTTCGGCTACCACTTATGGATGGACCCGCGGTGCATTAGTTAGTTGGTG<br>AGGTAATGGCTCACCAAGACCATGATGCATAGCCGACCTGAGAGGGTGATCGGCCACA<br>CTGGGACTGAGACACGGCCCAGACTCCTACGGGAGGCAGCAGTAGGGAATCTTCCGCA<br>ATGGACGAAAGTCTGACGGAGCAATGCCGCGTGAGTGAAGAAGGTTTTCGGATCGTAA<br>AACTCTGTTGTTAGAGAAGAACAAGGATGAGAGTAACTGCTCATCCCCTGACGGTATC<br>TAACCAGAAAGCCACGGCTAACTACGTGCCAGCAGCCGCGGTAATACGTAGGTGGCAA<br>GCGTTGTCCGGATTTATTGGGCGTAAAGCGAGCGCAGGCGGTTCTTTAAGTCTGATGTG<br>AAAGCCCCCGGCTCAACCGGGGAGGGTCATTGGAAACTGGAGAACTTGAGTGCAGAA<br>GAGGAGAGTGGAATTCCATGTGTAGCGGTGAAATGCGTAGATATATGGAGGAACACCA<br>GTGGCGAAGGCGACTCTCTGGTCTGTAACTGACGCTGAGGCTCGAAAGCGTGGGGAGC<br>AAACAGGATTAGATACCCTGGTAGTCCACGCTGTAAACGATGAGTGCTAAGTGTTGGA<br>GGGTTTCCGCCCTTCAGTGCTGCAGCTAACGCATTAAGCACTCCGCCTGGGGAGTACGA<br>CCGCAAGGTTGAAACTCAAAGGAATTGACGGGGACCCGCACAAGCGGTGGAGCATGT<br>GGTTTAATTCGAAGCAACGCGAAGAACCTTACCAGGTCTTGACATCCTTTGACCACTCG<br>AGAGATCGAGCTTTCCCTTCGGGGACAAAGTGACAGGTGGTGCATGGTTGTCGTCAGC<br>TCGTGTCGGAA	1055	99.9%	99%	*Carnobacterium divergens*	NR_113798.1
456	E2	T12	PCA	CATGCAGTCGAGCGGACTTAAAAAGCTTGCTTTTTAAGTTAGCGGCGGACGGGTGAGT<br>AACACGTGGC	68	98.5%	98%	*Bacillus nealsonii*	NR_044546.1
515	E2	T12	VRBGA	GGCGGCGGGCCTACCACATGCAAGTCGAGCGGATGAGAGGAGCTTGCTCCTCGATTCA<br>GCGGCGGACGGGTGAGTAATGCCTAGGAATCTGCCTAGTAGTGGGGGACAACGTTTCG<br>AAAGGAACGCTAATACCGCATACGTCCTACGGGAGAAAGTGGGGGATCTTCGGACCTC<br>ACGCTATTAGATGAGCCTAGGTCGGATTAGCTAGTTGGTAGGGTAAAGGCCTACCAAG<br>GCGACGATCCGTAACTGGTCTGAGAGGATGATCAGTCACACTGGAACTGAGACACGGT<br>CCAGACTCCTACGGGAGGCAGCAGTGGGGAATATTGGACAATGGGCGAAAGCCTGATC<br>CAGCCATGCCGCGTGTGTGAAGAAGGCCTTCGGGTCGTAAAGCACTTTAAGTTGGGAG<br>GAAGGGCTCATAGCGAATACCTGTGAGTTTTGACGTTACCAACAGAATAAGCACCGGC<br>TAACTTCGTGCCAGCAGCCGCGGTAATACGAAGGGTGCAAGCGTTAATCGGAATTACT<br>GGGCGTAAAGCGCGCGTAGGTGGCTTGATAAGTTGGATGTGAAATCCCCGGGCTCAAC<br>CTGGGAACTGCATCCAAAACTGTCTGGCTAGAGTGCGGTAGAGGGTAGTGGAATTTCC<br>AGTGTAGCGGTGAAATGCGTAGATATTGGAAGGAACACCAGTGGCGAAGGCGACTAC<br>CTGGACTGACACTGACACTGAGGTGCGAAAGCGTGGGGAGCAAACAGGATTAGATAC<br>CCTGGTAGTCCACGCCGTAAACGATGTCAACTAGCCGTTGGGATCCTTGAGATCTTAGT<br>GGCGCAGCTAACGCATTAAGTTGACCGCCTGGGGAGTACGGCCGCAAGGTTAAAACTC<br>AAATGAATTGACGGGGGCCCGCACAAGCGGTGGAGCATGTGGTTTAATTCGAAGCAAC<br>GCGAAGAACCTTACCTGGCCTTGACATGCTGAGAACTTTCCAGAGATGGATTGGTGCCT<br>TCGGGAACTCAGACACAGGTGCTGCATGGCTGTCGTCAGCTCGTGTCGTGAGATGTTG<br>GGTTAAGTCCCGTAACGAGCCCAACCCTTGTCCTTAGTTACCAGCACGTTATGGTGGGC<br>ACTCTAAGGAACTGCCGGTGACAACCGGAAGAAGGTGGGGATGACGTCAAGTCATCAT<br>GGCCTTACGGC	1172	99.4%	100%	*Pseudomonas* sp.	NR_114041.1

Strain ID	Product (tray)	Sample	Medium	Sequence	bp	% identity	Query coverage	Name	Accession n°
378	E3	T0	PCA	GGTGCTAATACATGCAAGTCGAACGCACTCTCGTTTAGATTGAAGGAGCTTGCTCCTGATTGATAAACATTTGAGTGAGTGGCGGACGGGTGAGTAACACGTGGGTAACCTGCCCTAAAGTGGGGGATAACATTTGGAAACAGATGCTAATACCGCATAAAACCTAACACCGCATGGTGTAGGGTTGAAAGATGGTTTCGGCTATCACTTTAGGATGGACCCGCGGTGCATTAGTTAGTTGGTGAGGTAAAGGCTCACCAAGACCGTGATGCATAGCCGACCTGAGAGGGTAATCGGCCACACTGGGACTGAGACACGGCCCAGACTCCTACGGGAGGCAGCAGTAGGGAATCTTCCACAATGGACGAAAGTCTGATGGAGCAACGCCGCGTGAGTGAAGAAGGTTTTCGGATCGTAAAACTCTGTTGTTGGAGAAGAATGTATCTGATAGTAACTGATCAGGTAGTGACGGTATCCAACCAGAAAGCCACGGCTAACTACGTGCCAGCAGCCGCGGTAATACGTAGGTGGCAAGCGTTGTCCGGATTTATTGGGCGTAAAGCGAGCGCAGGCGGTTTCTTAAGTCTGATGTGAAAGCCTTCGGCTCAACCGAAGAAGTGCATCGGAAACTGGGAAACTTGAGTGCAGAAGAGGACAGTGGAACTCCATGTGTAGCGGTGAAATGCGTAGATATATGGAAGAACACCAGTGGCGAAGGCGGCTGTCTGGTCTGTAACTGACGCTGAGGCTCGAAAGCATGGGTAGCAAACAGGATTAGATACCCTGGTAGTCCATGCCGTAAACGATGAGTGCTAGGTGTTGGAGGGTTTCCGCCCTTCAGTGCCGCAGCTAACGCATTAAGCACTCCGCCTGGGGAGTACGACCGCAAGGTTGAAACTCAAAGGAATTGACGGGGGCCCGCACAAGCGGTGGAGCATGTGGTTTAATTCGAAGCAACGCGAAGAACCTTACCAGGTCTTGACATCCTTTGACCACTCTAGAGATAGAGCTTTCCCTTCGGGGACAAAGTGACAGGTGGTGCATGGTTGTCGTCAGCTCGTGTCGTGAGATGTTGGGTTAAGTCCCGCAACGAGCGCAACCCTTATACTAGTTGCCAGCATTAATTGGGCACTCTA	1136	99.7%	99%	*Latilactobacillus sakei*	NR_113821.1
459	E3	T12	PCA	GGTGCTAATACATGCAAGTCGAACGCACTCTCGTTTAGATTGAAGGAGCTTGCTCCTGATTGATAAACATTTGAGTGAGTGGCGGACGGGTGAGTAACACGTGGGTAACCTGCCCTAAAGTGGGGGATAACATTTGGAAACAGATGCTAATACCGCATAAAACCTAACACCGCATGGTGTAGGGTTGAAAGATGGTTTCGGCTATCACTTTAGGATGGACCCGCGGTGCATTAGTTAGTTGGTGAGGTAAAGGCTCACCAAGACCGTGATGCATAGCCGACCTGAGAGGGTAATCGGCCACACTGGGACTGAGACACGGCCCAGACTCCTACGGGAGGCAGCAGTAGGGAATCTTCCACAATGGACGAAAGTCTGATGGAGCAACGCCGCGTGAGTGAAGAAGGTTTTCGGATCGTAAAACTCTGTTGTTGGAGAAGAATGTATCTGATAGTAACTGATCAGGTAGTGACGGTATCCAACCAGAAAGCCACGGCTAACTACGTGCCAGCAGCCGCGGTAATACGTAGGTGGCAAGCGTTGTCCGGATTTATTGGGCGTAAAGCGAGCGCAGGCGGTTTCTTAAGTCTGATGTGAAAGCCTTCGGCTCAACCGAAGAAGTGCATCGGAAACTGGGAAACTTGAGTGCAGAAGAGGACAGTGGAACTCCATGTGTAGCGTTGAAATGCGTAGATATATGGAAGAACACCAGTGGCGAAGGCGGCTGTCTGGTCTGTAACTGACGCTGAGGCTCGACAGCATGGGTAGCAAACAGGATTAGATACCCTGGTAGTCCATGCCGTAGACGATGAGTGCTAGGTGCTGGA	824	99.4%	99%	*Latilactobacillus sakei*	NR_113821.1
460	E3	T12	PCA	CTGCGGCAGCCTAACACATGCAAGTCGAGCGGTAACAGGAAGAAGCTTGCTTCTTTGCTGACGAGCGGCGGACGGGTGAGTAATGTATGGGGATCTGCCTGACAGAGGGGGATAACTACTGGAAACGGTAGCTAATACCGCATAATCTCTAAGGAGCAAAGCAGGGGACCTTCGGGCCTTGCGCTGTCGGATGAACCCATATGGGATTAGCTAGTAGGTGAGGTAATGGCTCACCTAGGCGACGATCTCTAGCTGGTCTGAGAGGATGATCAGCCACACTGGGACTGAGACACGGCCCAGACTCCTACGGGAGGCAGCAGTGGGGAATATTGCACAATGGGCGCAAGCCTGATGCAGCCATGCCGCGTGTATGAAGAAGGCCTTAGGGTTGTAAAGTACTTTCAGTCGGGAGGAAGGCGTTGATATTAATACTATCAGCGATTGACGTTACCGACAGAAGAAGCACCGGCTAACTCCGTGCCAGCAGCCGCGGTAATACGGGGGGTGCAAGCGTTAATCGGAATTACTGGGCGTAAAGCGCACGCAGGCGGTTGATTAAGTTAGATGTGAAATCCCCGGGCTTAACCTGGGAATGGCATCTAAAACTGGTCAGCTAGAGTCTTGTAGAGGGGGGTAGAATTCCATGTGTAGCGGTGAAATGCGTAGAGATGTGGAGGAATACCGGTGGCGAAGGCGGCCCCCTGGACAAAGACTGACGCTCAGGTGCGAAAGCGTGGGGAGCAAACAGGATTAGATACCCTGGTAGTCCACGCTGTAAACGATGTCGATTTGGAGGTTGTTCCCTTGAGGAGTGGCTTCCGGAGCTAACGCGTTAAATCGACCGCCTGGGGAGTACGGCCGCAAGGTTAAAACTCAAATGAATTGACGGGGGCCCGCACAAGCGGTGGAGCATGTGGTTTAATTCGATGCAACGCGAAGAACCTTACCTACTCTTGACATCCAGAGAATTTAGCAGAGATGCTTTAGTGCCTTCGGGAACTCTGAGACAGGTGCTGCATGGCTGTCGTCAGCTCGTGTTGTGAAATGTTGGGTTAAGTCCCGCAACGAGCGCAACCCTTATCCTTTGTTGCCAGCACGTCATGGTGGGAACTCAGGGAGACTGCCGGTGATAAACCGGAGGAAGGGGG	1148	99.5%	99%	*Moellerella wisconsensis*	NR_104939.1

Strain ID	Product (tray)	Sample	Medium	Sequence	bp	% identity	Query coverage	Name	Accession n°
461	E3	T12	PCA	GTGCTAATACATGCAAGTCGAACGCACTCTCGTTTAGATTGAAGGAGCTTGCTCCTGAT TGATAAACATTTGAGTGAGTGGCGGACGGGTGAGTAACACGTGGGTAACCTGCCCTAA AGTGGGGGATAACATTTGGAAACAGATGCTAATACCGCATAAAACCTAACACCGCATG GTGTAGGGTTGAAAGATGGTTTCGGCTATCACTTTAGGATGGACCCGCGGTGCATTAGT TAGTTGGTGAGGTAAAGGCTCACCAAGACCGTGATGCATAGCCGACCTGAGAGGGTAA TCGGCCACACTGGGACTGAGACACGGCCCAGACTCCTACGGGAGGCAGCAGTAGGGA ATCTTCCACAATGGACGAAAGTCTGATGGAGCAACGCCGCGTGAGTGAAGAAGGTTTT CGGATCGTAAAACTCTGTTGTTGGAGAAGAATGTATCTGATAGTAACTGATCAGGTAG TGACGGTATCCAACCAGAAAGCCACGGCTAACTACGTGCCAGCAGCCGCGGTAATACG TAGGTGGCAAGCGTTGTCCGGATTTATTGGGCGTAAAGCGAGCGCAGGCGGTTTCTTA AGTCTGATGTGAAAGCCTTCGGCTCAACCGAAGAAGTGCATCGGAAACTGGGAAACTT GAGTGCAGAAGAGGACAGTGGAACTCCATGTGTAGCGGTGAAATGCGTAGATATATGG AAGAACACCAGTGGCGAAGGCGGCTGTCTGGTCTGTAACTGACGCTGAGGCTCGAAAG CATGGATAGCAAACAGGATTAGATA	780	99.7%	100%	*Latilactobacillus sakei*	NR_113821.1
583	E3	T12	RBCA	AAAAAGACCTTACACACAGTGTTTTTTGTTATTACAAGAACTTTTGCTTTGGTCTGGAC TAGAAATAGTTTGGGCCAGAGGTTTACTGAACTAAACTTCAATATTTATATTGAATTGT TATTTATTTAATTGTCAATTTGTTGATTAA	148	99.3%	100%	*Debaryomyces* sp.	NR_077067.1

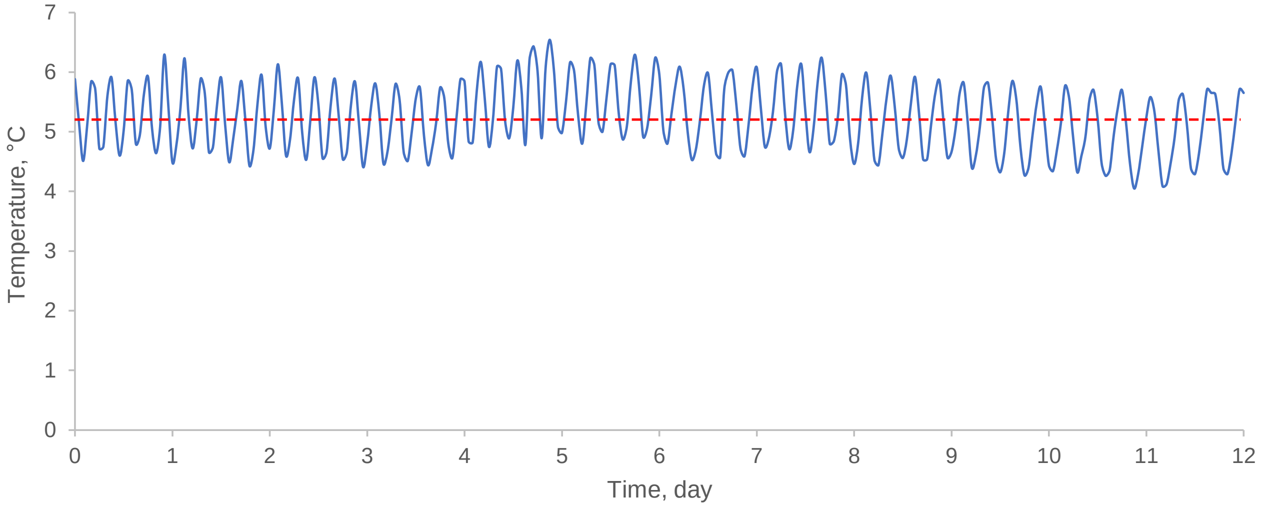

Supplementary Figure 1. Temperature profile recorded in the domestic refrigerator during the 12 days-storage of cooked ham samples. The red dashed line represents the mean value calculated across the whole monitoring period.

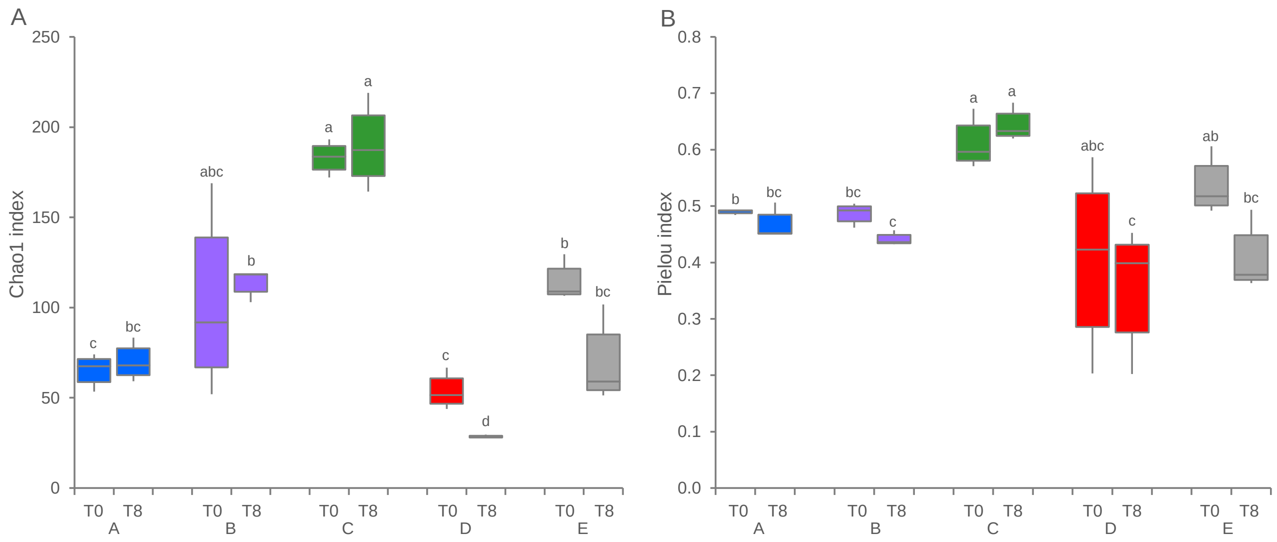

Supplementary Figure 2. Indexes of α diversity based on ASV identification in the coocked ham samples; Chao1, richness; Pielou, eveness. Common lowercase letters indicate non significant differences between values

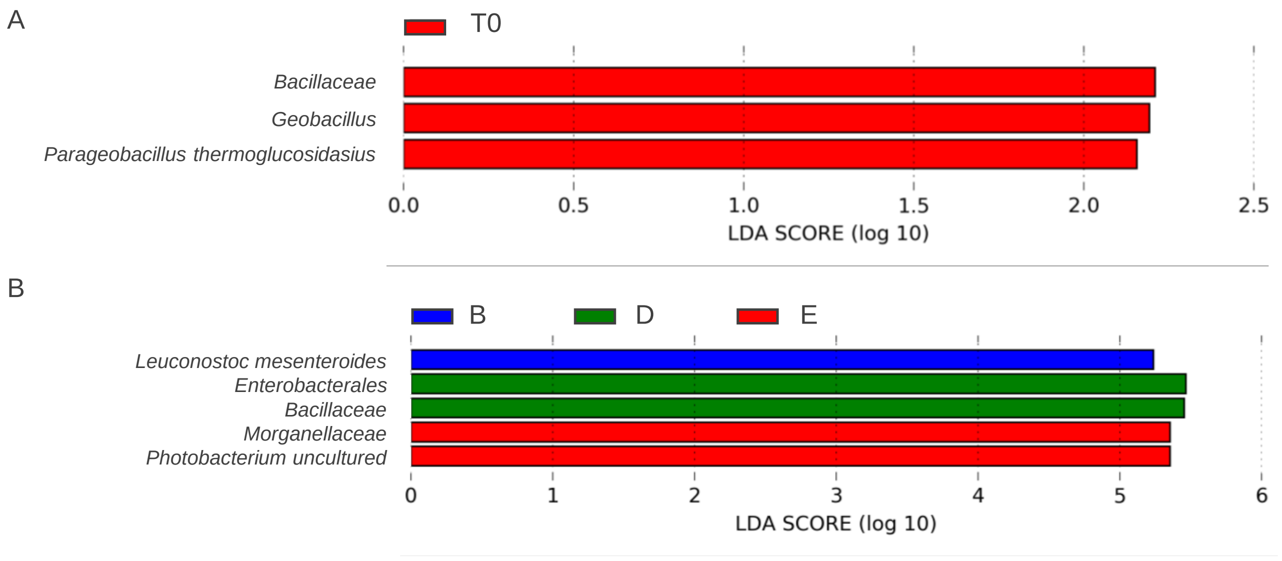

Supplementary Figure 3. Linear discriminant analysis Effect Size (LEfSe) of taxonomic features characterizing T0 samples or appearing as specific features of distinct product.

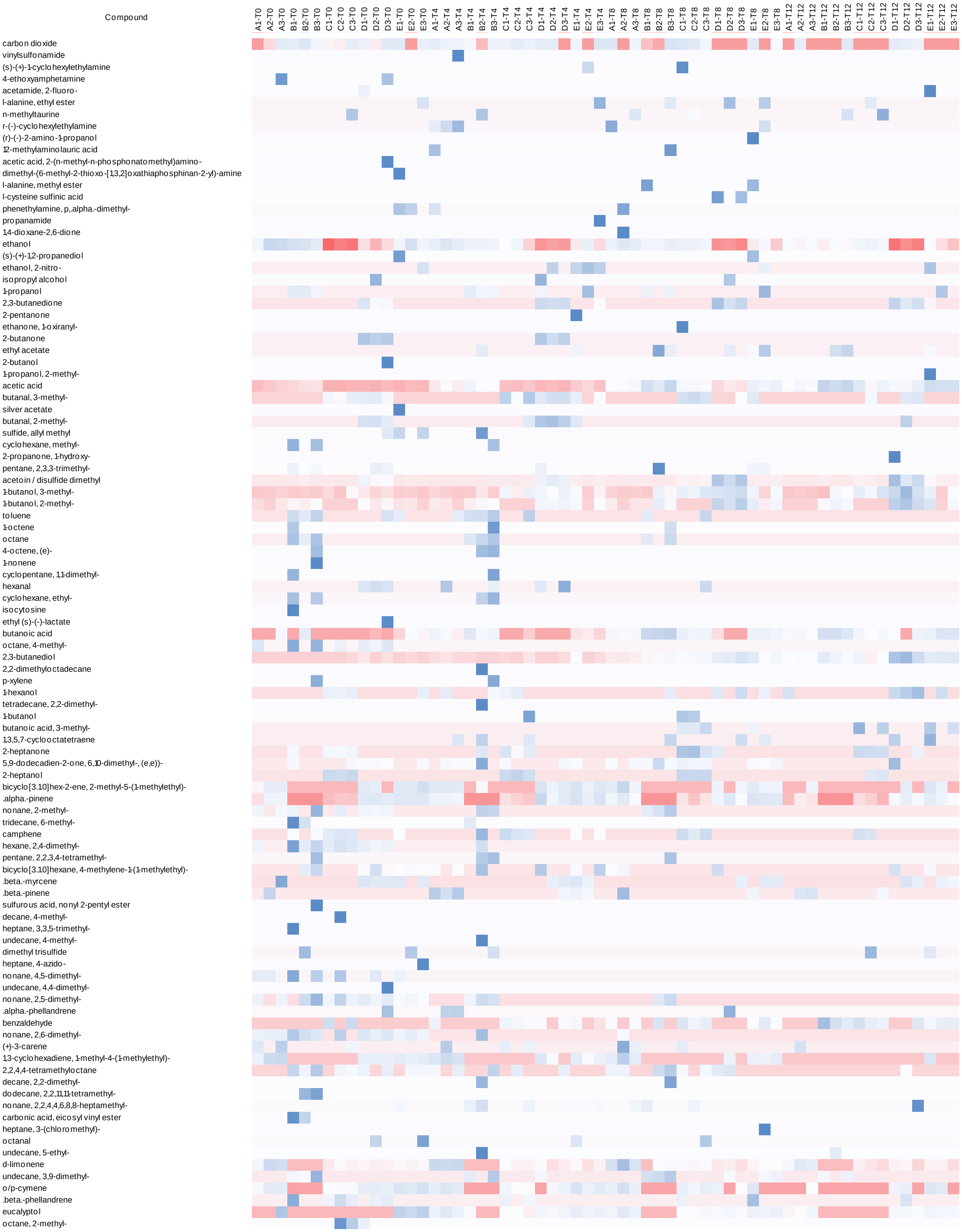
Compound
A1-T0
A2-T0
A3-T0
B1-T0
B2-T0
B3-T0
C1-T0
C2-T0
C3-T0
D1-T0
D2-T0
D3-T0
E1-T0
E2-T0
E3-T0
A1-T4
A2-T4
A3-T4
B1-T4
B2-T4
B3-T4
C1-T4
C2-T4
C3-T4
D1-T4
D2-T4
D3-T4
E1-T4
E2-T4
E3-T4
A1-T8
A2-T8
A3-T8
B1-T8
B2-T8
B3-T8
C1-T8
C2-T8
C3-T8
D1-T8
D2-T8
D3-T8
E1-T8
E2-T8
E3-T8
A1-T12
A2-T12
A3-T12
B1-T12
B2-T12
B3-T12
C1-T12
C2-T12
C3-T12
D1-T12
D2-T12
D3-T12
E1-T12
E2-T12
E3-T12
carbon dioxide
vinylsulfonamide
(s)-(+)-1-cyclohexylethylamine
4-ethoxyamphetamine
acetamide, 2-fluoro-
l-alanine, ethyl ester
n-methyltaurine
r-(-)-cyclohexylethylamine
(r)-(-)-2-amino-1-propanol
12-methylaminolauric acid
acetic acid, 2-(n-methyl-n-phosphonatomethyl)amino-
dimethyl-(6-methyl-2-thioxo-[1,3,2]oxathiaphosphinan-2-yl)-amine
l-alanine, methyl ester
l-cysteine sulfinic acid
phenethylamine, p,.alpha.-dimethyl-
propanamide
1,4-dioxane-2,6-dione
ethanol
(s)-(+)-1,2-propanediol
ethanol, 2-nitro-
isopropyl alcohol
1-propanol
2,3-butanedione
2-pentanone
ethanone, 1-oxiranyl-
2-butanone
ethyl acetate
2-butanol
1-propanol, 2-methyl-
acetic acid
butanal, 3-methyl-
silver acetate
butanal, 2-methyl-
sulfide, allyl methyl
cyclohexane, methyl-
2-propanone, 1-hydroxy-
pentane, 2,3,3-trimethyl-
acetoin / disulfide dimethyl
1-butanol, 3-methyl-
1-butanol, 2-methyl-
toluene
1-octene
octane
4-octene, (e)-
1-nonene
cyclopentane, 1,1-dimethyl-
hexanal
cyclohexane, ethyl-
isocytosine
ethyl (s)-(-)-lactate
butanoic acid
octane, 4-methyl-
2,3-butanediol
2,2-dimethyloctadecane
p-xylene
1-hexanol
tetradecane, 2,2-dimethyl-
1-butanol
butanoic acid, 3-methyl-
1,3,5,7-cyclooctatetraene
2-heptanone
5,9-dodecadien-2-one, 6,10-dimethyl-, (e,e))-
2-heptanol
bicyclo[3.1.0]hex-2-ene, 2-methyl-5-(1-methylethyl)-
.alpha.-pinene
nonane, 2-methyl-
tridecane, 6-methyl-
camphene
hexane, 2,4-dimethyl-
pentane, 2,2,3,4-tetramethyl-
bicyclo[3.1.0]hexane, 4-methylene-1-(1-methylethyl)-
.beta.-myrcene
.beta.-pinene
sulfurous acid, nonyl 2-pentyl ester
decane, 4-methyl-
heptane, 3,3,5-trimethyl-
undecane, 4-methyl-
dimethyl trisulfide
heptane, 4-azido-
nonane, 4,5-dimethyl-
undecane, 4,4-dimethyl-
nonane, 2,5-dimethyl-
.alpha.-phellandrene
benzaldehyde
nonane, 2,6-dimethyl-
(+)-3-carene
1,3-cyclohexadiene, 1-methyl-4-(1-methylethyl)-
2,2,4,4-tetramethyloctane
decane, 2,2-dimethyl-
dodecane, 2,2,11,11-tetramethyl-
nonane, 2,2,4,4,6,8,8-heptamethyl-
carbonic acid, eicosyl vinyl ester
heptane, 3-(chloromethyl)-
octanal
undecane, 5-ethyl-
d-limonene
undecane, 3,9-dimethyl-
o/p-cymene
.beta.-phellandrene
eucalyptol
octane, 2-methyl-

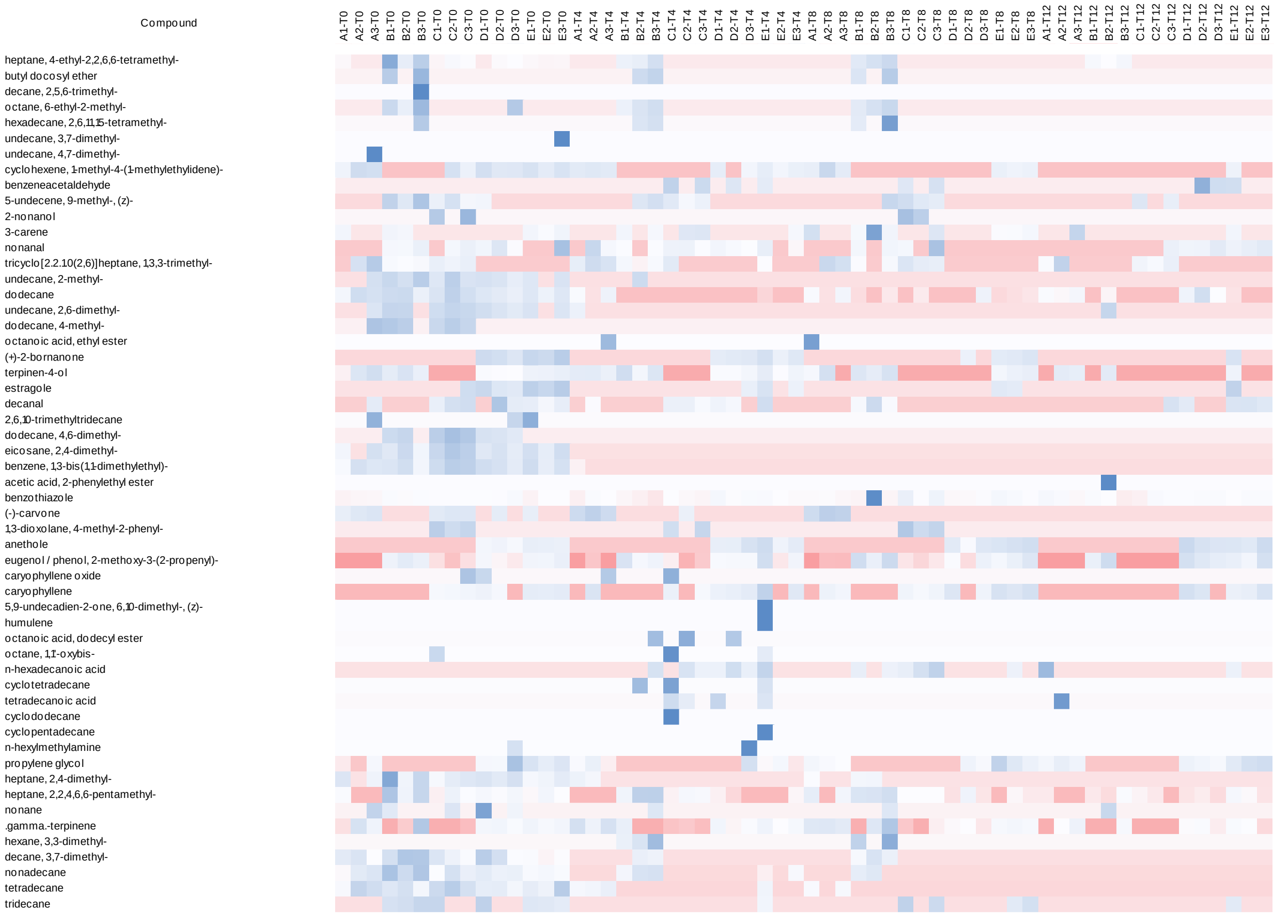

Supplementary Figure 4. Distribution among coocked ham samples of the 157 volatile organic compounds (VOCs), identified in the headspace of cooked ham samples during the secondary shelf life. The area of each VOC was meancentered and normalized by the standard deviation. Values are reported as colors ranging from the lowest (deepest blue) to the highest (deepest red).
